# *JAGGED* Controls *Arabidopsis* Petal Growth and Shape by Interacting with a Divergent Polarity Field

**DOI:** 10.1371/journal.pbio.1001550

**Published:** 2013-04-30

**Authors:** Susanna Sauret-Güeto, Katharina Schiessl, Andrew Bangham, Robert Sablowski, Enrico Coen

**Affiliations:** 1 Department of Cell and Developmental Biology, John Innes Centre, Norwich Research Park, Norwich, United Kingdom; 2 School of Computing Sciences, University of East Anglia, Norwich Research Park, Norwich, United Kingdom; Cambridge University, United Kingdom

## Abstract

A flowering plant generates many different organs such as leaves, petals, and stamens, each with a particular function and shape. These types of organ are thought to represent variations on a common underlying developmental program. However, it is unclear how this program is modulated under different selective constraints to generate the diversity of forms observed. Here we address this problem by analysing the development of Arabidopsis petals and comparing the results to models of leaf development. We show that petal development involves a divergent polarity field with growth rates perpendicular to local polarity increasing towards the distal end of the petal. The hypothesis is supported by the observed pattern of clones induced at various stages of development and by analysis of polarity markers, which show a divergent pattern. We also show that *JAGGED* (*JAG*) has a key role in promoting distal enhancement of growth rates and influences the extent of the divergent polarity field. Furthermore, we reveal links between the polarity field and auxin function: auxin-responsive markers such as DR5 have a broader distribution along the distal petal margin, consistent with the broad distal organiser of polarity, and *PETAL LOSS* (*PTL*), which has been implicated in the control of auxin dynamics during petal initiation, is directly repressed by *JAG*. By comparing these results with those from studies on leaf development, we show how simple modifications of an underlying developmental system may generate distinct forms, providing flexibility for the evolution of different organ functions.

## Abstract 2

Computational modeling and experimentation show how *Arabidopsis* petals develop their size and shape through a growth pattern that is distinct to, but operates within, the developmental framework that also controls leaf shape.

## Author Summary

Flowering plants generate organs with different shapes and functions. Leaves, for example, are photosynthetic organs, while petals provide a broad display that attracts pollinators. In spite of their diversity, these organs are thought to represent variations of a common underlying developmental program. It is unclear how this program is modulated to generate diverse forms, while also being subject to selective constraints. We address this issue by determining the growth rules that underlie petal shape and compare these rules to those for leaf development. We show that, in contrast to the leaf, which has a pattern of growth orientations that converges towards the leaf tip, the petal has a pattern of growth orientations that fans out. Nevertheless, both leaf and petal growth modes operate within the same basic framework, which involves an internal polarity field that has a specified pattern of growth rates that occur parallel or perpendicular to it. We also show that the petal growth pattern is under the control of JAGGED, which promotes growth rates in a particular region of the petal while also modulating tissue polarity. Our analysis suggests that differences in organ shape have evolved through the interplay between growth rules that are constrained to operate within a basic developmental framework and selective constraints that reflect organ function.

## Introduction

Plant organs with different functions, such as leaves and petals, can be subject to very different developmental and selective constraints. Leaves are sink organs (net importers of photoassimilates) at early developmental stages but then increasingly act as sources (net exporters of photoassimilates) as their photosynthetic competence increases [1],[2]. They are also subject to herbivory both as mature structures and while they grow [3]. By contrast petals play a major role in attracting pollinators and are generated at a later stage of the life cycle, when the plant has already built up the reserves and metabolic machinery necessary to satisfy reproductive competence. Petals serve as sinks throughout their growth and are less subject to herbivory than leaves [4]–[6]. In spite of these differences, leaves and petals are thought to arise through a common developmental programme [7],[8]. A key question is the manner in which this programme is modulated to generate particular leaf and petal forms under different selective constraints [9]–[11].

A particularly tractable system for addressing this issue is Arabidopsis. Leaves and petals of this species have a different shape: leaves are elliptical and narrow towards their distal tip, while petals broaden out distally to form a paddle shape. The development of Arabidopsis leaves has been extensively studied using clonal analysis, tracking, cell-cycle markers, and molecular genetics [12]–[18]. The cell polarity pattern of developing leaves has also been studied through the auxin efflux carrier PIN1, which localises to the distal end of epidermal cells. PIN1 polarity converges towards the leaf primordium tip [19]–[21]. The auxin response marker DR5 is strongly expressed at the distal leaf tip, consistent with auxin being transported towards it through the epidermis [19],[20],[22]. Polarity of internal tissue appears to be oriented in the opposite direction, with PIN1 being localised to the proximal end of cells in vascular and provascular tissue [19]–[21]. This pattern corresponds to internalised auxin being transported away from the distal leaf tip towards the base.

Based on the above findings, a working hypothesis for leaf morphogenesis has been proposed [15]. This hypothesis invokes a polarity field that converges towards the distal tip of the leaf, mirroring the observed PIN polarity pattern. Leaf shape then arises through a specified pattern of growth rates parallel and perpendicular to the polarity field. Growth rates parallel to the polarity field decline towards the tip of the leaf, correlating with early differentiation of the distal region and continued growth in the proximal (basal) region [12],[15]–[18].

In contrast to the Arabidopsis leaf, little is known about the polarity pattern and growth dynamics of the Arabidopsis petal [23]–[28]. The distribution of PIN proteins has not been described for petal primordia, and expression of DR5 has only been described for initiating petals, when it defines the centre of the anlagen [29],[30]. Genetic experiments suggest that the transcription factor encoded by *PETAL LOSS* (*PTL*) functions upstream of auxin-mediated signalling in the control of petal initiation [30]. Different stages of petal development have been defined based on landmarks of flower development [24] and petal size [27],[31], but growth tracking or clonal analysis on Arabidopsis petals has not been performed. Several genes have been shown to affect petal shape, including *JAGGED* (*JAG*) and *FRILL1* [26],[32]–[34]. The petals of the *frill1* mutant have a frilled distal margin. *FRILL1* encodes a sterol methyltransferase (*STM2*) that suppresses endoreduplication in the distal part of the petal [33],[34]. The petals of the *jag* mutant are narrow and have a reduced distal region with a jagged or serrated margin [26],[32]. *JAG* enodes a zinc finger transcription factor expressed during the emergence of all shoot organs [26],[32],[35]. *JAG* is expressed in the distal domain of the petal [26],[32]. Based on reduced cell-cycle activity in *jag-1* petals (as assessed by expression of histone H4), *JAG* function has been linked to cell proliferation during organ growth [26]. Recently *JAG* has been identified as a key regulator of the transition from meristems to sepal primordia identity, including a shift from isotropic to anisotropic growth, increased cell proliferation, and cell enlargement [35]. However, it is unclear how *JAG* influences petal shape.

Here we use a combination of experimental (clonal analysis, molecular genetic analysis, imaging of polarity markers) and computational modelling approaches to determine how Arabidopsis petals acquire their shape. In contrast to leaf development, which is thought to involve a convergent polarity field and reduced growth rates towards the distal tip, we propose that petal development involves a divergent polarity field, with growth rates perpendicular to local polarity increasing towards the distal end of the petal. The divergent polarity field may arise through a broad organiser of tissue polarity along the distal margin of the developing petal, which acts as an attractor of polarity. The hypothesis is supported by the observed pattern of clones induced at various stages of development and by analysis of polarity markers that show a divergent pattern. In addition, auxin-responsive markers such as DR5 have a broader distribution along the distal petal margin, consistent with a broad distal organiser. We also show that *JAG* is likely to be a key gene involved in promoting distal enhancement of growth rates and the extent of the divergent polarity field. This role for *JAG* is supported by the *JAG* expression pattern, the *jag* mutant petal phenotype, and the effect of ectopic *JAG* on petal shape. We also show that *JAG* directly represses *PTL*, suggesting that the effect of *JAG* on the polarity field may be mediated by regulating auxin dynamics via *PTL*. We conclude that together with changes in the pattern of specified growth rate, changes in orientations of the polarity field provide a simple mechanism by which diversity in organ shape may be generated in plants.

## Results

### Clonal Analysis of Petal Development

**Figure 1:**
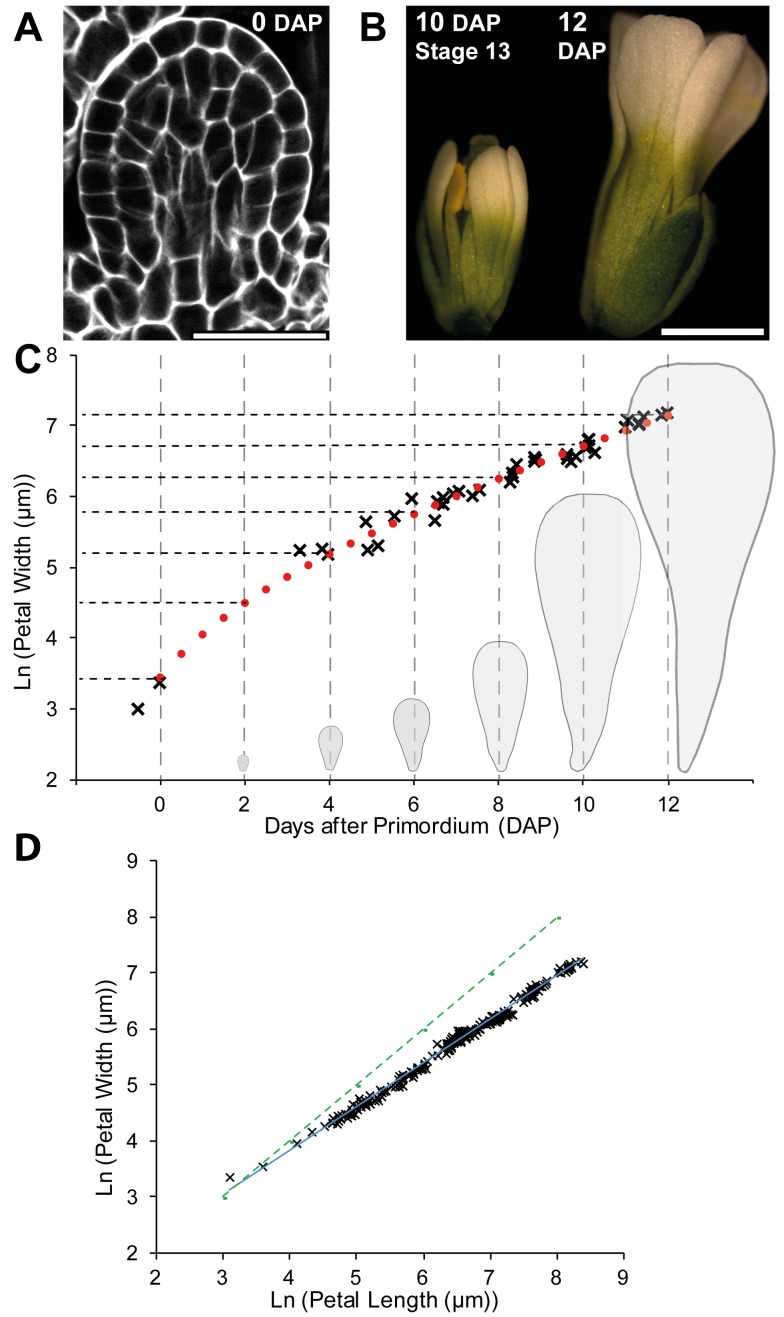
Petal growth analysis. (A) Section of a petal primordium around day 0 DAP (days after primordium formation) (width, 35 µm). (B) Petals at 10 DAP and 12 DAP (some sepals overlying the petals have been removed). (C) Standard flower 1 petal width (natural logarithm scale) against time (black asterisks) and fitted logistic curve (red dotted line) from initiation until maturity; time in days after primordium formation (DAP). The gradient of the curve is the growth rate in width. Dashed lines indicate average petal width at 2-d interval as estimated with the fitted curve: ∼30 µm (0 DAP), 90 µm (2 DAP), 180 µm (4 DAP), 315 µm (6 DAP), 520 µm (8 DAP), 820 µm (10 DAP), and 1,265 µm (12 DAP). These time points can be related to previously defined flower stages [24] as follows: stages 5–6 (before 0 DAP), stages 7–9 (between 0–2 DAP), stage 10 (between 4–6 DAP), stages 11–12 (between 6–8 DAP), and stage 13 (10 DAP). (D) Petal length plotted against width during petal development (natural logarithm scales). The gradient of the fitted line is 0.78, showing that growth rate in width is less than in length. For comparison, the dotted green line has a gradient of 1. Scale bar, 20 µm (A), 1 mm (B).

A convenient starting point for our analysis is when petal primordia have as simple shape and are about 30 µm wide. We refer to this stage as 0 DAP (0 d after primordium formation) (Figure 1A). Petals took about 12 d to grow from 0 DAP to maturity, by which time they had an elongated shape that was wider towards the distal end (Figure 1B). At the end of the growth period, petals detach from the flower (abscission). Based on logarithmic plots of maximum petal width (at the petal's widest point, which is at the distal part of the petal) against time, we estimated that the growth rate in width was about 1.8% h^−1^ during 0–4 DAP and then about 1% h^−1^ for the remaining duration of petal development (Figure 1C). Plots of petal width against length showed that the growth rate in width is about 80% of the growth rate in length (Figure 1D). The maximum areal growth rate at earlier stages (0–4 DAP) is ∼4.1% h^−1^ (1.8% h^−1^ in width and 2.3% h^−1^ in length) and at later stages (4–12 DAP) is ∼2.3% h^−1^ (1% h^−1^ in width and 1.3% h^−1^ in length). The growth rates could also be captured with a smooth curve (Figure 1C, red dotted line). Based on the above growth analysis, we divided petal development into a series of 2-d intervals and estimated the average width of the petals at these times, which were used as a subsequent framework in our study (gray dashed lines, Figure 1C).

**Figure 2:**
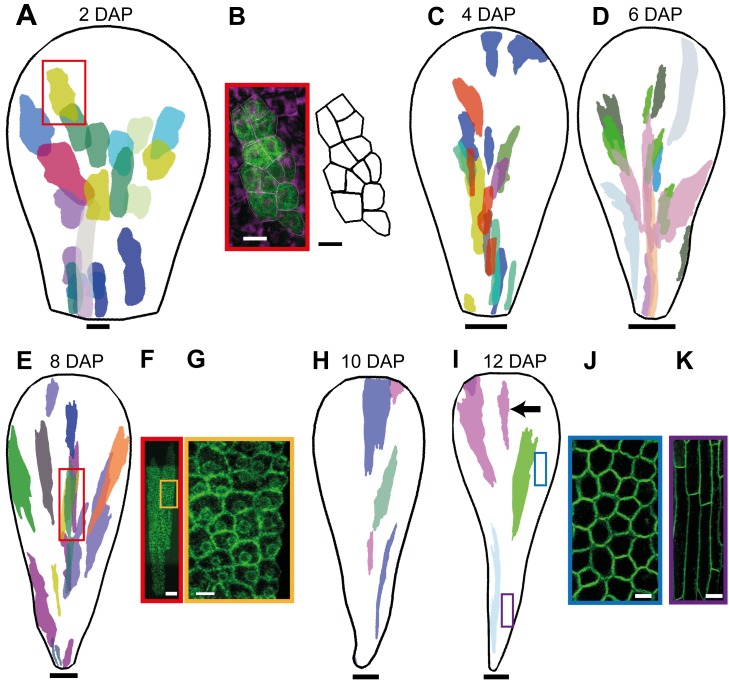
Petal clonal analysis after induction at 0 DAP. (A) Sector map with shapes and positions of clones induced at 0 DAP and imaged 2 d after induction (at a width that corresponds to 2 DAP). Clones were imaged from dissected, flattened petals and warped to an average petal shape and overlaid (using a different colour for each petal). (B) Image of a distal epidermal clone (framed with a red box in A) and the corresponding outline of cells. (C) Sector map with clones induced at 0 DAP and imaged 4 d after induction (at a width that corresponds to 4 DAP). (D) Sectors imaged at 6 d (6 DAP). (E) Sectors imaged at 8 d (8 DAP). (F) Image of a distal epidermal clone (framed with a red box in E). (G) Enlargement of the yellow box in (F) showing some epidermal cells. (H) Sector map with clones induced at 0 DAP and imaged at 10 d after induction (10 DAP). (I) Sectors imaged at 12 d (12 DAP). The black arrow points to a distal clone. (J) Distal epidermal cells at 12 DAP at the position of the blue box in (I). (K) Proximal epidermal cells at the position of the violet box in (I). Images at (A, C–E, H, I) are scaled to the same length to allow the difference in clone shape to be compared. Scale bar, 5 µm (B, G), 10 µm (A, J), 20 µm (F, K), 50 µm (C), 100 µm (D, E), 200 µm (H), and 300 µm (I).

To analyse growth patterns from 0 DAP onwards, we induced sectors in petal primordia at 0 DAP and visualised them at the end of each 2-d interval (i.e., 2, 4, 6, 8, 10, 12 DAP). This was achieved using a heat-shock-inducible Cre-Lox system. We induced sectors in a line carrying *hsp18.2::Cre* and *35S::lox-uidA-lox-GFP* [36]. A ∼1.5-min heat shock was given about 24 d after sowing, when the oldest flower was about to open. We then left the plants to grow for 2, 4, 6, 8, 10, and 12 d and imaged sectors in petals with widths that correspond to 2–12 DAP, respectively. At each imaging stage, epidermal and subepidermal sectors from several petals were collated and mapped onto an average petal shape to produce a combined sector map (Figure 2). This allows clones initiated at 0 DAP to be visualised at successively later stages.

The results show that clones have an anisotropic shape from the earliest stages visualised and become increasingly anisotropic at later stages (Figure 2). For example, for petals at 2, 4, 8, and 12 DAP, clones in the distal half have a length-to-width ratio (L/W) of about 2, 4, 7, and 7, respectively. For the proximal region, the L/W values are about 3, 5, 7, and 11. The increasing L/W ratio of clones with stage of visualisation is consistent with anisotropic growth being maintained for an extended period. For example, if a clone grows at ∼2.5% h^−1^ along its length and at 1% h^−1^ along its width, then over 2 d the L/W ratio of a clone would increase by a factor of ∼2, while over 4 d it would increase by ∼4. Thus we estimate that growth rate is about twice as high along the main axis of the clones compared to the minor axis for early stages of development. The degree of anisotropy may decline in the distal half of the petal after 8 DAP as the L/W of the clones does not change very much after this time.

Anisotropic growth of clones is associated with different cell division rates along the major axis of the clone. For example, at 2 DAP epidermal clones in the distal half were about 4–6 cells long and 2–3 cells wide, indicating ∼2 rounds of cell division along the major axis of the clone, and ∼1 round along the minor axis (Figure 2B). At 8 DAP epidermal distal clones were about 30–60 cells long and 4–10 cells wide, indicating that from 0 DAP there had been ∼5–6 rounds of cell division along the major axis and 2–3 rounds of division along the minor axis (Figure 2F).

Changes in cell shape also made a contribution to anisotropic growth in some regions, particularly at later stages of development when cell division rate decreased. Based on the cell numbers in the epidermal clones, cell division rates were highest from 0–4 DAP and then decreased, particularly from 8 DAP onwards for the distal region (Table S1), in good agreement with cell division rates previously described in the petal [27],[31]. Nevertheless, areal growth rate of the petal was maintained at ∼2.3% h^−1^ (Figure 1C), indicating that cells continued to enlarge at a similar rate even though cell divisions were less frequent. This is reflected in an increase in cell area: the average cell area of epidermal cells in the distal part of the petal at 2, 4, 6, 8, 10, and 12 DAP was ∼20 µm^2^, 30 µm^2^, 35 µm^2^, 40 µm^2^, 120 µm^2^, and 325 µm^2^, respectively (Figure 2B,F,G,J), which is in good agreement with cell size development described in the petal [27],[37]. The large increase in area at 10 DAP is consistent with cell divisions slowing down around this time. The L/W ratio for cells at later stages (12 DAP) was ∼1.2 for distal cells. This shows that anisotropy of growth occurred primarily in association with cell division for distal regions. For proximal regions the L/W ratio for epidermal cells at 12 DAP was ∼6–7 (Figure 2K), showing that anisotropic growth in proximal regions continued after cell divisions slowed down, leading to highly elongated cells [25],[28].

In addition to the degree of anisotropy, the orientation of the major axes of the clones provides information on growth patterns. In proximal regions, the major axis of the clones was oriented parallel to the midline, while in the distal half the orientations tended to fan out. The fanning out was evident from the earliest stage of clone visualisation (Figure 2A), indicating that growth was oriented in this fashion from early on.

**Figure 3:**
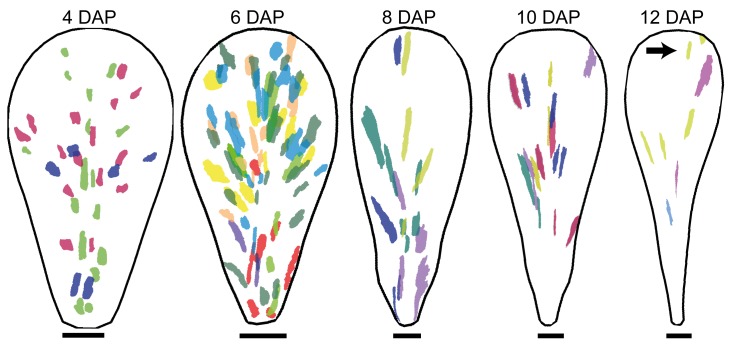
Petal clonal analysis after induction at 2 DAP. Sector map with shapes and positions of clones induced at 2 DAP and imaged 2, 4, 6, 8, or 10 d after induction (at widths that correspond to 4–12 DAP, respectively). For each image stage, petals are dissected and flattened, clones imaged, warped to an average petal shape, and overlaid (using a different colour for each petal). The black arrow points to distal clones at 12 DAP. Scale bar, 50 µm (4 DAP), 100 µm (6, 8 DAP), 200 µm (10 DAP), and 300 µm (12 DAP).

The above analysis gives the cumulative growth of clones induced at 0 DAP, but to estimate growth of clones initiated later, we performed equivalent experiments by inducing clones at 2 DAP (Figure 3). This was achieved by heat shocking plants as before but visualising clones at stages that correspond to 2 d after those in the previous experiment. Thus for a plant that grew for 2 d after heat shock, clones were visualised at the 4 DAP stage. These clones thus derive from primordia that were at the 2 DAP stage during heat shock rather than 0 DAP under the previous experiment. Similarly, clones from plants that grew for 4, 6, 8, and 10 d after heat shock were visualised at 6, 8, 10, and 12 DAP, respectively.

Again, clones were highly anisotropic and fanned out particularly in distal regions (Figure 3). Some fanning out towards the lateral edges was also evident for lateral proximal clones (Figure 3, 6 and 8 DAP), and may relate to a bulge in petal width at the base of the petals during these stages.

Based on the above clonal analysis, we conclude the following: (1) Growth is distributed over the whole petal as clones do not vary greatly in size from one region to another. (2) Growth rates are higher at earlier stages of petal development, during which growth is largely attended by cell division. At later stages, cell division rates fall, although growth continues through cell enlargement. (3) Growth is anisotropic for an extended period, with clones having a major axis of elongation from the earliest stages, and becoming more anisotropic as they grow for longer periods. (4) The major axis of the clones tends to be oriented proximodistally but fans out particularly in more distal regions.

### Petal Development Can Be Accounted for by a Divergent Polarity Field

We used the results from clonal analysis to evaluate hypotheses for petal growth. The aim of this study was not to provide a detailed model that would account for all aspects of petal growth, but to compare broad classes of models to determine whether they could account for the overall transformations in petal shape and clonal patterns. These models were formulated using the Growing Polarised Tissue framework (GPT framework), in which growth rates can be specified by a distribution of factors over a tissue [15],[38],[39]. Regions of the tissue are mechanically connected, forming a canvas, allowing the deformation resulting from specified local growth patterns to be computed. Each model has three components: an initial canvas shape with distributed factors, a system for specifying polarity, and a growth regulatory network.

**Figure 4:**
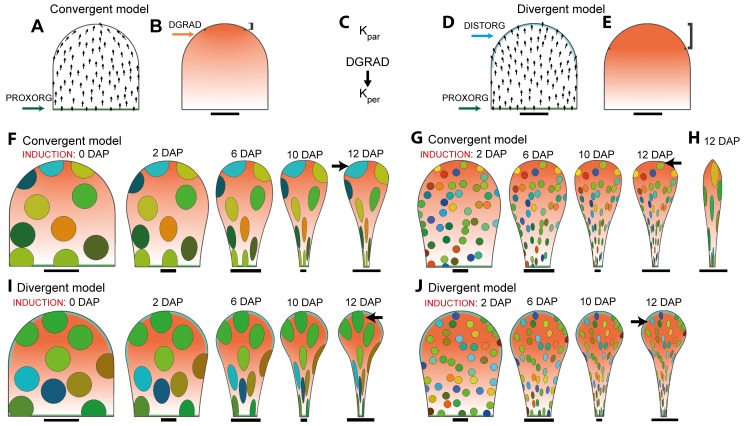
Model for petal development with a convergent or a divergent polarity field. (A) Canvas at 0 DAP for convergent model. Polarity field (black arrows) depends on a proximodistal gradient of a factor POL generated at the PROXORG region (in green) and degraded everywhere at a constant rate. (B) Distribution of DGRAD (terracotta) for convergent model (bracket indicates plateau of maximum DGRAD). (C) Regulatory network for the convergent and the divergent model. K_par_ has a fixed value, whereas K_per_ is promoted by DGRAD. (D) Canvas at 0 DAP for divergent model. Compared to (A) POL is now degraded at a high rate at the DISTORG (in cyan) region. The result is a field of polarities that diverges toward the distal margin. (E) Distribution of DGRAD (terracotta) for divergent model (bracket indicates plateau of maximum DGRAD). (F) Output of convergent model for 0–12 DAP, showing pattern of virtual clones induced at 0 DAP. All outputs scaled to same length. (G) Same as (F) but virtual clones induced at 2 DAP. (H) Output of convergent model in which promotion of K_per_ by DGRAD is reduced as compared to (F). Canvas is at 12 DAP, with virtual clones induced at 0 DAP. (I) Output of divergent model for 0–12 DAP, showing patterns of virtual clones induced at 0 DAP. (J) Same as (I) but virtual clones induced at 2 DAP. Black arrows point to distal clones at 12 DAP in (F, G, I, J). Scale bar, 10 µm (A, B, D, E, F) (0–2 DAP), (G) (2 DAP), (I) (0–2 DAP), (J) (2 DAP); 100 µm (F, G, I, J) (6–10 DAP); 1 mm (F–J) (12 DAP).

The starting shape for the canvas is based on a simplified petal primordium shape at 0 DAP (Figures 1A and 4A). Given the observed anisotropy of clones, we used models that incorporate tissue polarity, which allows orientations of preferential growth to be specified locally. According to this framework, growth orientations are specified by the gradient of a factor, POLARISER (POL), which can propagate through the canvas. POL is generated at regions referred to as +organisers and may also be degraded at a high rate in regions referred to as −organisers. The gradient of POL provides a local polarity that enables two types of growth rate to be specified through the growth regulatory network: growth rate parallel to the polarity axis (K_par_) and growth rate perpendicular to the polarity axis (K_per_). The pattern of specified growth rates leads to deformation of the canvas. The resultant growth pattern and shape may contain features, such as curvature, that were not directly specified as they arise through the constraints of tissue connectedness [39],[40].

We first developed a model for petal development that incorporates a tissue polarity pattern similar to that employed in a previously published model for leaf development (Figure 4A) [15]. In this model, POL production is promoted at the base of the canvas through an identity factor PROXORG (+organiser) and is degraded everywhere at a constant rate. Propagation of POL through the canvas generates a proximodistal field of polarities that is parallel to the midline in the basal half of the initial canvas and converges towards the tip (the position most distant from the +organiser) (Figure 4A, black arrows). We refer to this model as the *convergent model*.

The observed anisotropy of petal clones suggests that K_par_ should be higher than K_per_. This was implemented in our model by assuming a basic value for K_par_ of 1.7% h^−1^ and K_per_ of 0.65% h^−1^ throughout the petal. To account for the broadening shape of the petal towards the distal region, we also invoked a factor DGRAD, which has a value of 0 at the base of the canvas and increases linearly to a plateau value of 1 shortly before the distal tip (Figure 4B). DGRAD promotes K_per_, as shown in the growth regulatory network (Figure 4C), and the distribution of DGRAD deforms together with the tissue during growth (i.e., it is fixed to tissue).

The output of this model was a canvas with a final length, width, and outline shape similar to that of a mature petal (Figure 4F, 12 DAP). To determine whether the growth pattern also matched experimental data, we created virtual clones by marking circular regions on the initial canvas (equivalent to 0 DAP) and monitoring their shapes as they deform with the canvas (Figure 4F). Similar to experimental clones, virtual clones were anisotropic in proximal regions of the petal and showed a tendency to fan out more distally. However, the virtual clones were almost isotropic in shape towards the distal end of the petal at later stages (arrow in Figure 4F, 12 DAP) in contrast to experimental clones that are highly anisotropic (arrow in Figure 2I). The same problem (i.e., round distal clones at late stages) was observed for virtual clones induced at 2 DAP (compare arrowed clones in Figure 4G, 12 DAP with Figure 3, 12 DAP). The round distal clone shape is generated because of the requirement of high rates of K_per_ in the distal domain, which ensure that the petal becomes wider distally. If we reduce the extent by which DGRAD promotes K_per_, clones are more anisotropic distally but the petal shape is now too narrow (Figure 4H).

To resolve this discrepancy in clone shapes between model outputs and experimental data, we explored an alternative hypothesis in which polarity does not converge at the tip but fans out towards the distal margin. This was implemented by using a broad distal organiser (DISTORG, −organiser) that degrades POL at a high rate (Figure 4D). We refer to this as the *divergent model*. To maintain a higher level of anisotropy in distal clones, compared to the previous model, we reduced the extent to which DGRAD promotes K_per_ and increased the basic value of K_par_ to 1.8% h^−1^. In addition, we expanded the distal region where DGRAD forms a plateau as this gives a better final petal shape (Figure 4E).

As with the convergent model, the final overall canvas shape and size generated by the divergent model showed a reasonable match to that of the wild-type petal (Figure 4I). In addition, the shape of virtual clones in the divergent model gave a much better match to observed clones. Distal clones were now elongated, similar to the experimental data (compare Figure 4I, 12 DAP with Figure 2I, 12 DAP). Moreover, virtual clones induced at 2 DAP fanned out more, similar to what is observed experimentally (compare arrowed clones in Figure 4J, 12 DAP with Figure 3, 12 DAP). Thus, the divergent model, in which there is a broad distal organiser as well as a proximal organiser, gives a better fit to all of the data than the convergent model.

### Testing Predictions of a Divergent Polarity Field and a Broad Distal Organiser

**Figure 5:**
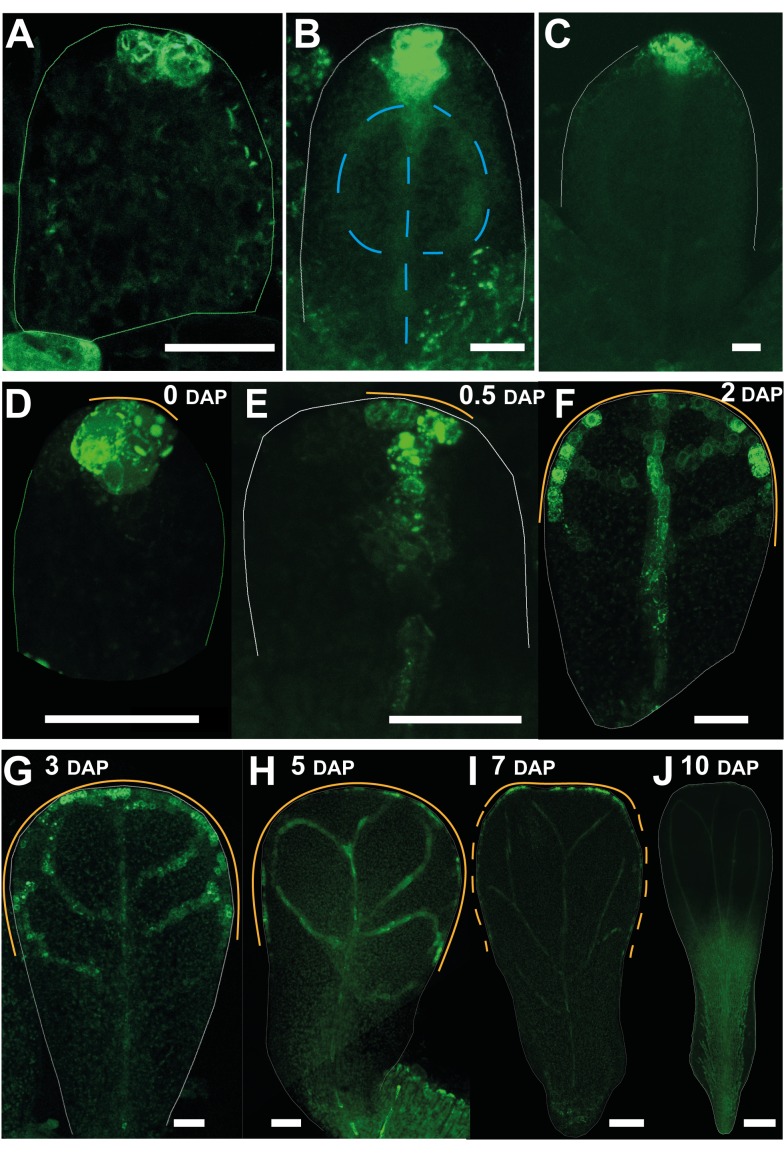
Broad distal distribution of auxin response marker *DR5::GFP* in the petal distal margin. (A–C) In leaf primordia, DR5 is expressed at the tip through early to later stages of development. DR5 is also expressed in the internal provasculature system (marked with a broken blue line in B). (D, E) In petal primordia at 0 and 0.5 DAP, DR5 is expressed at the distal tip. The orange line indicates the extent of the DR5 signal along the petal margin. (F, G) At 2–3 DAP, DR5 activity expands to a broader distal domain of the petal margin. (H) At 5 DAP the broad pattern of petal marginal expression is maintained. (I) At 7 DAP petal marginal expression narrows (the broken orange line marks the region where the DR5 domain first disappears). (J) By 10 DAP petal marginal expression has disappeared. DR5 signal in the internal provasculature system of the petal in (E–H). Width of leaves (A–C): 46, 80, and 150 µm. Width of petals (D–J): 26, 45, 82, 141, 261, 374, and 875 µm. Scale bar, 20 µm (A–G), 40 µm (H), 80 µm (I), and 250 µm (J).

As a further test of these models, we analysed the distribution of factors that might mark the proposed distal organiser. Perhaps the best candidate for a molecule involved in polarity propagation in plants is auxin. In leaf primordia, auxin is thought to flow proximodistally in the epidermis and accumulate at the distal tip, consistent with the convergent model. From there it flows back towards proximal regions through subepidermal and provascular tissue, defining the position of the future midvein ([19], Figure 5B, central broken blue line). The epidermal polarity pattern is supported by a major site of auxin accumulation at the leaf tip, as indicated by expression of the auxin response marker *DR5::GFP* (Figure 5A–C).

We therefore imaged the *DR5::GFP* marker [41],[42] in developing petals to determine whether they have a site of epidermal auxin accumulation at the distal tip or whether they have a broader domain of accumulation, as might be expected from the divergent model. At early stages of petal development (0–0.5 DAP), signal was seen at the distal tip of the petal primordia (orange line, Figure 5D,E), similar to the pattern observed in leaf primordia at a similar size (Figure 5A). However, at slightly later stages (1–3 DAP) signal was seen over a broader distal domain of the petal marginal epidermis (orange line, Figure 5F,G), although the signal was not uniform but varied in intensity along the margin. By contrast, leaf primordia at a similar size continued to show signal in a more restricted region at the distal tip (Figure 5B,C). The extended distal expression of DR5 in petals is consistent with epidermal polarity diverging towards a broad distal −organiser. DR5 signal was also seen in the internal provascular system of the petal primordia, and may reflect auxin transport from the epidermal distal organiser towards the petal interior (Figure 5E–H). The broad pattern of distal epidermal expression was maintained in petal primordia during later stages (3–6 DAP, Figure 5H), although it eventually narrowed (6–10 DAP, Figure 5I) and then disappeared (10 DAP, Figure 5J). Thus, the DR5 analysis is consistent with auxin in the petal epidermis being transported to a broad distal region for much of development as expected from the divergent model in which petals have a broad distal organiser.

**Figure 6:**
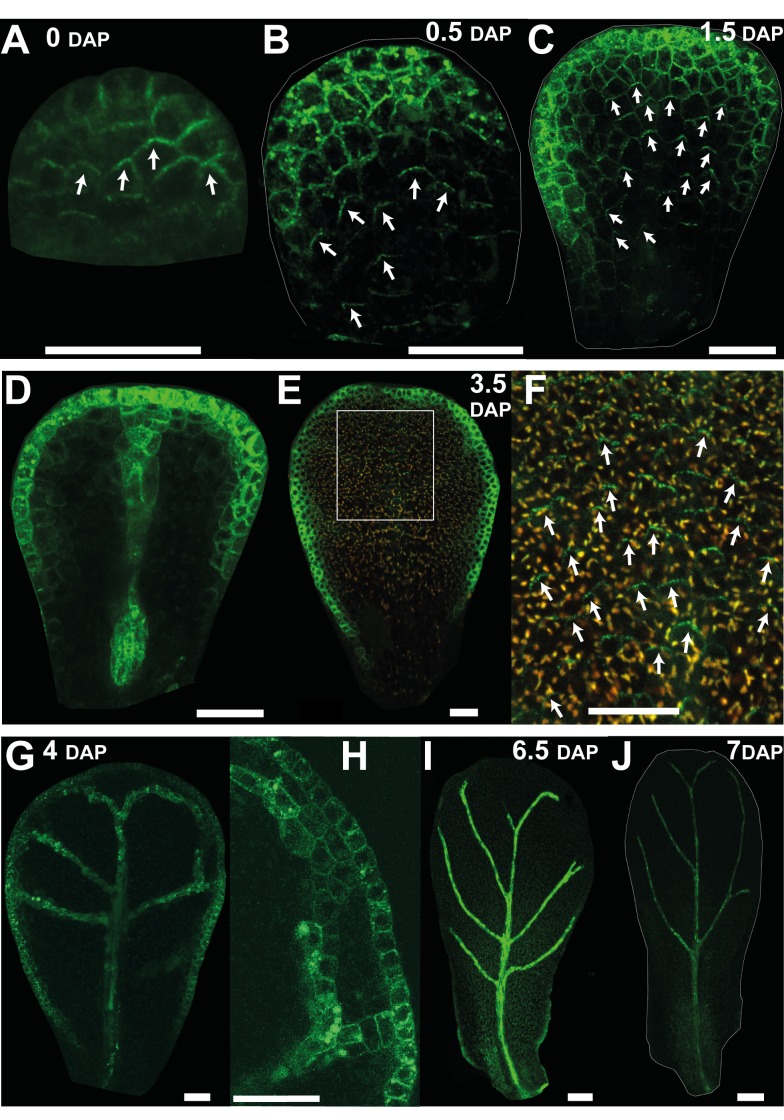
Epidermal expression pattern of the auxin efflux carrier PIN1 in petals supports a divergent polarity field. (A) At 0 DAP, epidermal PIN1::GFP is preferentially localised to the distal end of cells in the plane of the petal. White arrows on cells point towards the centre of the distal PIN1 expression. (B, C) At 0.5 and 1.5 DAP, PIN1 polarity in the epidermis points divergently toward the distal end of the tissue. Strong expression is also seen at or near the petal margin but without clear polar localisation. (D) Deeper section of (C) showing expression in the provascular tissue. (E) At 3.5 DAP, PIN expression in the main plain of the epidermis is weak. (F) Enlargement of the white box in (E) showing PIN1 signal at the distal end of the epidermal cells. PIN1 expression is lower so the GFP channel is shown merged with the red channel (corresponding to the emission of chlorophyll autofluorescence) to facilitate visualisation. (G) At 4 DAP, PIN1 signal is no longer detected in the main plane of the petal but can be observed in the petal margin and vascular tissue. (H) Close up of (G). (I) At 6.5 DAP, expression at the distal margin has disappeared. (J) Little signal is observed from 7 DAP onwards. Width of petals: (A–C) 30, 45, and 75; (E) 146; (G) 170; (I) 343; (J) 425 µm. Scale bar, 20 µm (A–H), 50 µm (I), and 80 µm (J).

A further marker for cell polarity is the auxin efflux carrier protein PIN1, which localises to the distal end of epidermal cells in early leaf primordia [19],[20]. We therefore monitored the pattern of PIN1 expression in developing petal primordia using a *PIN1::PIN1:GFP* fusion [41]. At 0 DAP epidermal PIN1 was preferentially localised to the distal end of cells in the plane of the organ (Figure 6A). This distal location was particularly evident in cases where PIN1 spanned cell junctions. To clarify the polarity pattern, we drew arrows on cells pointing toward the centre of the distal PIN1 signal when it clearly spanned a cell junction (white arrows Figure 6A). At 0.5 DAP the orientation of polarity suggests that it points divergently rather than convergently towards the distal end of the tissue (Figure 6B). Strong expression is also seen at or near the petal margin but without clear polar localisation. At 1.5 DAP the divergent pattern of polarity in the main plane of the epidermis was still evident (Figure 6C). Strong expression was also seen in the provascular and margin tissue at this stage (Figure 6D). At 3.5 DAP, epidermal signal was lower in the main plane of the petal but could still be seen with high laser exposure and had a divergent pattern (Figure 6E,F). Strong signal was again observed in the petal margin at this stage but without clear polar localisation. Between 4 and 7 DAP, PIN1 signal could no longer be detected in the main plane of the petal but could still be observed in the petal margin and vascular tissue (Figure 6G–I). Little signal was observed from 7 DAP onwards (Figure 6J). Thus, the results from this analysis indicate that epidermal cells in the main plane of the petal have divergent polarity pattern from 0.5–3.5 DAP, after which the PIN1 marker could not be detected in these cells. This is again consistent with the divergent model, and indicates that maintenance of polarity after 3.5 DAP may involve proteins other than PIN1.

**Figure 7:**
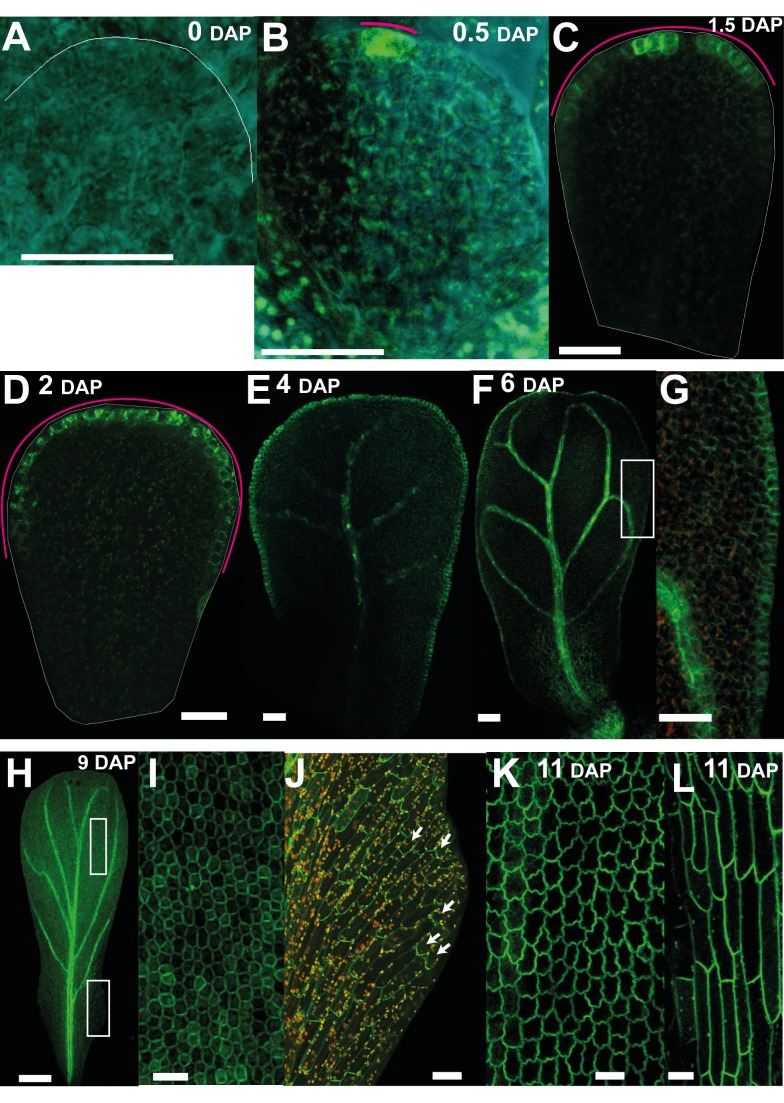
Auxin efflux carrier PIN3 expression correlates with the distal organiser position. (A) At 0 DAP, *PIN3::PIN3:GFP* signal is not detected at early stages of petal development. (B) By 0.5 DAP, PIN3 expression is detected at the distal tip of the petal. The magenta line indicates the extent of the PIN3 signal. (C, D) At 1.5 and 2 DAP, expression extends all around the distal marginal domain in a continuous way. (E) At 4 DAP, expression is also seen at the proximal marginal domain and in the veins. (F) At 6 DAP, PIN3 also starts being expressed in the distal epidermis, particularly in the regions adjacent to the margin with preferential localisation at the top and the bottom cell walls. (G) Enlargement of the white box in (F). (H–J) At 9 DAP, PIN3 is expressed in the epidermis and vasculature. (I) Enlargement of the white box in the distal region in (H) showing that PIN3 is not polarly localised. (J) Enlargement of the white box in the proximal region in (H) showing that PIN3 polarity points away from the margin and proximally. (K–L) At 11 DAP, PIN3 signal is strong throughout the epidermis (in both distal regions, K, and proximal regions, L). Width of petals: (A–F) 30, 47, 68, 98, 196, and 330; (H) 658; and (K) 1,020 µm. Scale bar, 20 µm (A–E, G, I, K, L), 120 µm (F), and 200 µm (H).

In addition to PIN1, we found that PIN3 was also expressed in petal primordia from 0.5 DAP (Figure 7A,B). At this stage *PIN3:PIN3::GFP* reporter [43] gave a strong signal at the distal tip of the petal (magenta line, Figure 7B), similar to the DR5 reporter. At slightly later stages (1–3 DAP), PIN3 signal was in a broader distal domain (magenta line, Figure 7C,D), similar to the expression of DR5. At 4–6 DAP the PIN3 domain extended all around the petal margin (Figure 7E–G). It also started being expressed in the distal epidermis, especially in the regions adjacent to the margin with preferential localisation at the top and the bottom cell walls (Figure 7E–G). Expression was also detected in the veins (Figure 7E–H). From 8 DAP onwards PIN3 signal became stronger throughout the epidermis of the petal and was not polarly localised in the distal half of the petal (Figure 7I). In the proximal half of the petal, PIN3 signal was polarly localised in the epidermis with polarity pointing away from the margin (Figure 7J, white arrows). At later stages of petal development (11 DAP) the signal was strong throughout the epidermis (Figure 7K). Thus, PIN3 expression seems to correlate with the position of the putative −organiser as well as revealing a divergent polarity pattern at later stages.

### JAG Is a Candidate for a Distal Regulator of Growth

**Figure 8:**
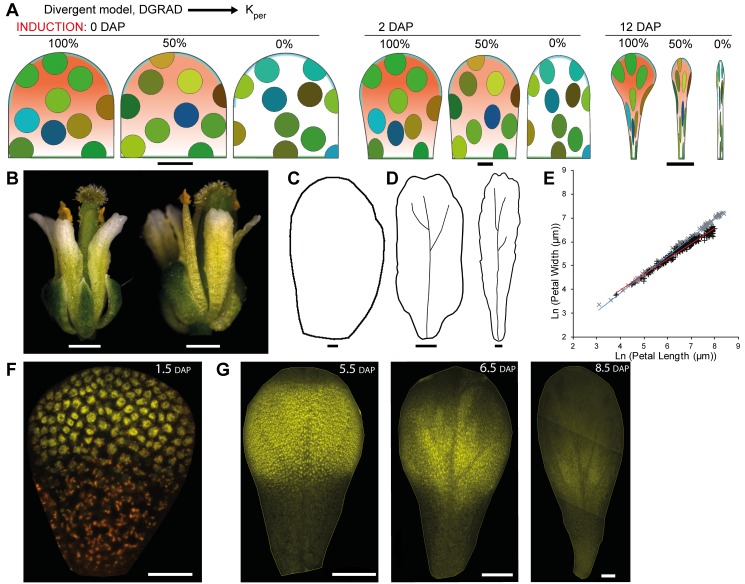
*JAGGED* is a good candidate for a distal-specific factor that contributes to DGRAD and promotes K_per_. (A) Canvas for divergent petal models with different DGRAD (terracotta region) activity in promoting K_per_. 100% activity corresponds to the wild-type model (as in Figure 4C–E), and 50% and 0% activity correspond to virtual mutant models in which the level of DGRAD is reduced to 50% or eliminated. Canvas is shown at 0 DAP (time of virtual clone induction), 2 DAP, and 12 DAP. (B) Flowers of *jag-2* and *jag-1* mutants. (C–D) Representative outlines of *jag-1* petals at various stages of development. Width of petals shown: 87, 310, and 530 µm. (E) Petal length against width throughout petal development (in natural logarithm scale) for wild type (gray crosses) and *jag-1* (black crosses). The blue and red lines are the linear regressions for the wild type and mutant, respectively. The gradient for *jag-1* is 0.65 compared to 0.78 for wild-type petals, showing that *jag* petals grow less in width compared to length (see also Figure 1D). (F–G) *JAG* expression pattern at various stages of petal development as visualised with the *jag-3* mutant line complemented with a *JAG::JAG:VENUS* construct. The widths for each DAP images are: 1.5 DAP (75 µm), 5.5 DAP (271 µm), 6.5 (363 µm), and 8.5 DAP (575 µm). For the image at 1.5 DAP the YFP channel is shown merged with the red channel (corresponding to the emission of chlorophyll autofluorescence) Scale bar, 10 µm (A) (0–2 DAP) (C), 20 µm (F), 100 µm (D, G), 500 µm (B), 1 mm (A) (12 DAP).

In addition to invoking particular organiser patterns, the models described above invoke a factor DGRAD, which has high levels in distal regions of the petal and promotes K_per_. The role of DGRAD in petal shape can be illustrated by creating virtual mutants in which the level of DGRAD is reduced to 50% or eliminated (Figure 8A). For both the convergent and divergent models, this gives petals that are much narrower (only the results for the divergent model are shown in Figure 8A). Petals are also slightly shorter, which arises because polarity is oriented diagonally in much of the distal region, with the consequence that the component of K_par_ contributing to length is reduced whilst the contribution from K_per_ is increased, decreasing petal length. Candidate genes that may contribute to DGRAD activity would thus be expected to give mutants with narrow, short petals and should be expressed at a higher level in distal regions of wild-type petals.

One such gene is *JAGGED* (*JAG*), which encodes a zinc finger transcription factor and gives short narrow petals in putative null mutants (Figure 8B–D; [26],[32]). Compared to wild type, in which growth rate in width is ∼80% of that in length, for *jag-1* mutant the growth rate in width is ∼70% that in length, consistent with reduced K_per_ (Figure 8E). Further support for a role of JAG in DGRAD activity is that it is expressed in the distal domain of the petal, as revealed by in situ hybridizations to longitudinal sections of flower buds [26]. To get a clearer picture of *JAG* expression in the plane of the petal, we used a *jag* mutant line complemented with a *JAG::JAG:VENUS* construct [32]. This showed that JAG is localised in the distal half of the petal from early stages (e.g., 1.5 DAP, Figure 8F). This expression pattern is maintained for several more days (5.5 DAP, Figure 8G), although signal declines in the most distal regions. The decline in signal spreads proximally (6.5 DAP, Figure 8G), and eventually signal becomes very weak throughout the petal (8.5 DAP, Figure 8G). Thus, based on both its mutant phenotype and expression pattern, *JAG* is a good candidate for a distal-specific factor that contributes to DGRAD and promotes K_per_.

**Figure 9:**
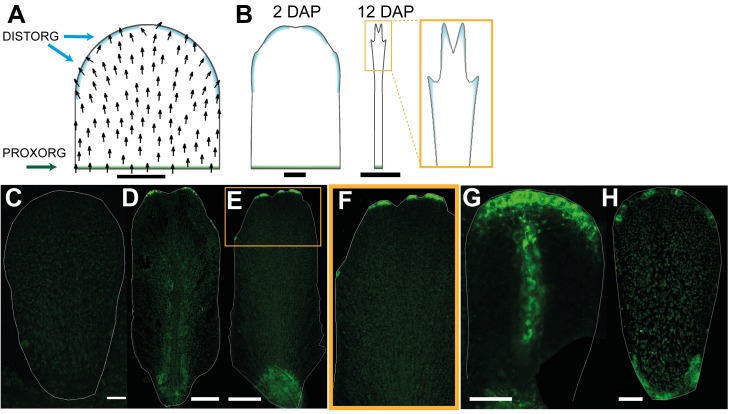
A less active and more discontinuous distal organiser in *jagged* mutants. (A) Initial canvas for divergent petal model (as in Figure 4D) with no DGRAD activity (as in Figure 8A, 0% model) and with a more discontinuous distal organiser activity as implemented by a discontinuous DISTORG (cyan) region. (B) Canvas at 2 and 12 DAP. Petals develop a jagged or serrated distal petal outline, with the tip of the serrations corresponding to regions with high DISTORG activity (as shown in the enlargement of the orange square at 12 DAP). (C) *DR5::GFP* signal is not detected at early stages in *jag-1* mutant, and (D, E) it is detected at later stages in several regions of the distal domain in a discontinuous way. (F) Enlargement of the orange square in (E). (G) *PIN1::PIN1:GFP* in *jag-1* mutant. PIN1 signal is reduced in the *jag-1* mutant at all stages and has a narrower distribution in the distal margin compared to wild-type petal (compare with Figure 6D). (H) *PIN3::PIN3:GFP* in *jag-1* mutant shows reduced expression, and it is more discontinuous in the distal margin (compare with wild type in Figure 7D). Width of petals: (C–G) 123, 310, 515, 66, and 84 µm. Scale bar, 10 µm (A, B) (2 DAP), 20 µm (C, G, H), 100 µm (D), 200 µm (E).

As *JAG* is expressed in distal petal regions, it also provides a candidate for a direct or indirect modulator of the broad distal organiser in the divergent model. For example, the distribution of the distal organiser could be promoted by *JAG* expression near the petal margin. An effect of *JAG* on the distal −organiser may also help to explain another feature of *jag* mutant petals: they have serrated or jagged distal margins (Figure 8D). This phenotype might be accounted for if *jag* mutants have a reduced and more discontinuous distal organiser activity. This would lead to a jagged or serrated distal outline phenotype, with the tip of the serrations corresponding to regions with high organiser activity (Figure 9A,B).

To test this possibility, we determined the pattern of *DR5::GFP*, *PIN1::PIN1:GFP*, and *PIN3::PIN3:GFP* expression in the *jag* mutant. Signal from the DR5 construct could not be detected at early stages in *jag* mutant petal primordia (Figure 9C). At later stages, when the *jag* petal primordia were more than half their mature width, DR5 signal started to be detected and was observed in several regions of the distal domain (Figure 9D–F). Thus, if DR5 signal is a marker for −organiser position, the organiser is reduced and more discontinuous in the *jag* mutant. Expression of PIN1 was reduced in the *jag* mutant at all stages and had a narrower distribution in the distal margin (compare Figure 9G with Figure 6D). The PIN1 expressing marginal region corresponds to where distal serrations form in the *jag* mutant. Expression of PIN3 was also reduced in the *jag* mutant (compare Figure 9H with Figure 7D). As with DR5, PIN3 signal was more discontinuous in the distal margin, although this pattern was observable at an earlier stage (compare Figure 9H with Figure 9C). Thus, PIN3 signal may also be a marker for −organiser activity, and its distribution confirms that the −organiser is less active and more discontinuous in the *jag* mutant.

### Test of Model through JAG Ectopic Expression

**Figure 10:**
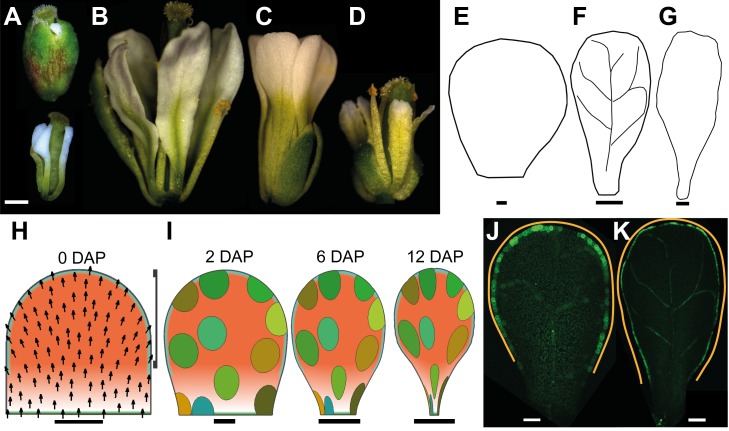
Effect of extending the DGRAD and distal organiser domains. (A) Ectopic *JAG* line (*AP1>>JAG*) flower with a strong phenotype in which sepal organs are fused and petal organs are reduced or deformed (same flower is shown with or without sepals). (B) Ectopic *JAG* line flower with an intermediate phenotype in which petals have a different shape, as if the region of the petal with distal identity has extended more proximally. (C) Wild-type flower with paddle shape petals. (D) *jag-1* mutant flower with petals with a reduced distal region. (E–G) Representative petal outlines from intermediate phenotype lines at various stages of development. Width of petals: 115 µm (E), 294 µm (F), 940 µm (G). (H) Initial canvas for divergent petal model with a proximally extended DGRAD region (terracotta, bracket indicates plateau of maximum DGRAD), and an extended DISTORG (cyan) region. The polarity field diverges at more proximal positions than in the wild-type model (compare with Figure 4D,E). (I) Model output at 2, 6, and 12 DAP showing patterns of virtual clones induced at 0 DAP and broadening of more proximal regions compared to the model for wild type (Figure 4J). (J, K) *DR5::GFP* expression in the intermediate phenotype ectopic *JAG* line extends more proximally compared with wild type (Figure 5G–I). The orange line indicates the extent of the DR5 signal. Width of petals: 139 µm (J), 294 µm (K). Scale bar, 10 µm (E, H, I) (2 DAP), 20 µm (J), 50 µm (K), 100 µm (F, I) (6 DAP), 200 µm (G), 500 µm (A–D), 1 mm (I) (12 DAP).

As a further way of testing whether *JAG* contributes to DGRAD and the distal organiser, we analysed lines ectopically expressing *JAG* under the control of the flower-specific promoter AP1 (line *AP1>>JAG*), which is active in the whole petal primordium until later stages of petal development (Figure S1). In addition to the previously described strong phenotypes in which sepal organs are fused and petal organs are reduced or deformed (Figure 10A) [26],[32], we obtained multiple lines with weaker *JAG* expression in which sepals were not fused and petals were wider (Figure 10B,E–G). The severity of the phenotypes correlated positively with the level of *JAG* expression (Figure S2). In plants with intermediate phenotypes, petals had a phenotype opposite to that of the *jag* mutant: the distal region of the petals, characterised by conical epidermal cells, was enlarged in comparison to the wild type (Figure 10B–D).

This intermediate phenotype could be readily accounted for by our model if we assume that *JAG* expression is extended more proximally in these lines (Figure 10H). This would lead to enhancement of DGRAD in proximal regions and a broader distal organiser (through the promoting effect of *JAG* on the distal organiser) (Figure 10H). The result is a petal in which the distal domain extends more proximally, resembling the ectopic *JAG* lines (Figure 10I). To determine whether the distal organiser has a broader distribution in the ectopic *JAG* line, we crossed the line to the DR5 marker line. As predicted, the distal marginal domain of *DR5* expression extended more proximally in the ectopic *JAG* line (Figure 10J,K, orange lines, compare with Figure 5G–I).

### *JAG* Directly Regulates *PTL*

A possible mechanism by which *JAG* enhances distal organiser activity is through regulation of *PTL*, which functions upstream of auxin-mediated signalling in the control of petal development [30]. *PTL* is expressed in the margin of the developing petal but excluded from the distal margin [44], where we propose the broad distal organiser acts. This exclusion may arise through *JAG* repressing *PTL* in this region, thus promoting −organiser activity.

**Figure 11:**
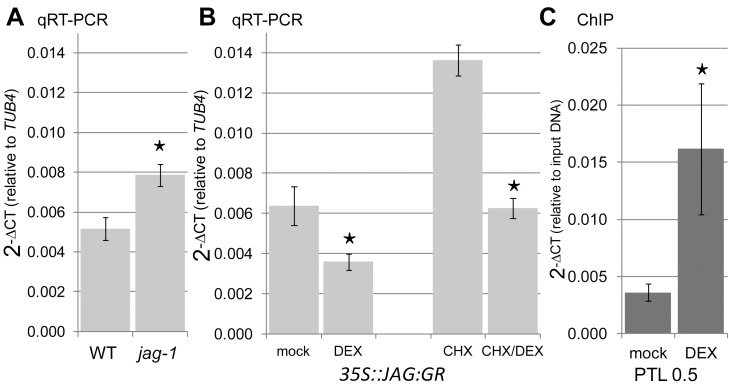
*JAGGED* directly represses *PETAL LOSS.* Expression levels (relative to the *TUB4* constitutive control) of *PTL* mRNA measured by qRT-PCR in (A) wild-type and *jag-1* inflorescence apices and (B) *35S::JAG:GR* inflorescence apices 5 h after mock treatment, treatment with dexamethasone 10 µm (DEX), cycloheximide 10 µm (CHX), or cycloheximide 10 µm combined with dexamethasone 10 µm (CHX/DEX). (C) Chromatin immunoprecipitation (ChIP) using anti-GR antibodies and inflorescence apices of *35S::JAG:GR* plants 4 h after mock treatment or treatment with DEX 10 µm. Target sequence 0.5 Kb upstream of the *PTL* start codon shows significant enrichment. Mu-like negative control did not show a significant difference (unpublished data). (A–C) Bars show the average and standard deviation of three biological replicates; asterisks indicate statistically significant differences (unpaired two-sample Student's *t* test, *p*<0.05) between the untreated wild type and *jag-1* (A) and between DEX-treated samples and corresponding controls (B, C).

To test whether *JAG* represses *PTL*, we first checked the levels of *PTL* mRNA by qRT-PCR in wild-type and *jag-1* inflorescences and confirmed that they were increased in the mutant compared with wild type (Figure 11A). We then determined whether *PTL* was directly down-regulated by *JAG* using a dexamethasone-inducible *35S::JAG:GR* system [35]. The results showed that activation of JAG:GR lowered *PTL* mRNA levels, and that this repression did not require new protein synthesis (Figure 11B). Chromatin immunoprecipitation (ChIP) using *35S::JAG:GR* inflorescences confirmed direct, dexamethasone-dependent binding of *JAG-GR* to the *PTL* promoter region (Figure 11C). We conclude that *JAG* acts as a direct transcriptional repressor of *PTL*.

## Discussion

In contrast to leaf development, which involves a convergent polarity field and reduced growth rates towards the distal tip, we show that petal development arises through a divergent polarity field with growth rates increasing distally. The divergent polarity field is implemented by having a broad organiser of tissue polarity along the distal margin of the petal primordium, which acts as an attractor of polarity. Specified growth rates perpendicular to the local polarity increase toward the distal end of the petal, giving a petal outline and clonal patterns similar to those observed experimentally. We estimate that relative growth rate is about twice as high along the main axis of the clones compared to the minor axis for early stages of development. This anisotropic growth is reflected in the pattern of cell divisions: cells proliferate more along the proximodistal axis than perpendicular to it. An alternative model, in which the polarity field converges distally, can also generate a good match to the observed petal outline. However, this convergent model is inconsistent with the observed patterns of clones. Thus, clonal patterns provide a more stringent test for underlying models than overall organ shape and suggest that unlike leaves, the petals of *Arabidopsis* have a broad distal organiser.

The proposed divergent polarity field is further supported by analysis of markers of auxin transport and response in the petal epidermis. Initially PIN1 is localised at the distal end of petal epidermal cells, but it then becomes localised more laterally, consistent with a divergent polarity pattern. This is in contrast to the distribution of PIN1 in leaf primordia, which shows a more consistent convergent pattern [19],[20]. The auxin response marker, *DR5::GFP*, and the PIN3 transporter are also enhanced in a broad region of the distal petal margin, consistent with the proposed location of a distal organiser. DR5 expression and PIN3 may thus be direct or indirect markers of organiser activity. This view is consistent with auxin playing a critical role in organisation of tissue polarity [41],[45].

Several models have been proposed for how auxin may influence cell polarity, through cell–cell comparison of auxin levels [46]–[48], measurement of auxin efflux [49]–[51], response to stress gradients induced by auxin [52], response to auxin concentration differences across the thickness of cell walls [53], and indirect cell–cell coupling mediated by auxin [54]. The nature of + or − organisers and their role in coordinating polarity has also been discussed in a cellular context [54]. The models for petal development presented here do not incorporate cellular mechanisms for polarity coordination as they treat tissue as a continuum. This framework provides a convenient abstraction to help simulate deformations at a tissue scale. It would be valuable to incorporate cellular mechanisms for polarity coordination within such a framework to provide a more integrated understanding of polarity coordination at different levels.

A key feature of our model is that growth rate perpendicular to the polarity field, K_per_, is enhanced in distal regions of the petal. The transcription factor *JAG* most likely contributes to this distal enhancement. *JAG* is expressed in the distal petal domain and *jag* mutants have narrow strap-like petals, as expected for a distal factor that promotes K_per_ [26],[32]. Moreover, lines that ectopically express *JAG* in petals have broader proximal regions, consistent with ectopic *JAG* promoting growth perpendicular to the local polarity.

The effect of *JAG* in promoting perpendicular growth in petals is in contrast to its effect in initiating sepal primordia, in which *JAG* enhances growth principally parallel to the proximodistal axis [35]. In *jag* mutants, sepal primordia grow almost isotropically at these very early stages. This growth change is observed at stages earlier than those described in this article and in regions that will eventually form the distal tip of the organ [55]. It is not clear whether the different effects of *JAG* on sepal compared to petal growth reflect differences in organ identity or the stage at which growth is monitored. In either case, *JAG* is proposed to have a differential effect on growth along different cellular axes for both petals and sepals. This suggests that *JAG* influences targets that may interact differentially with cell walls, weakening or strengthening walls in one orientation relative to another, and thus modulating the degree of anisotropy [56],[57]. According to our model, this orientation would be determined by local polarity, which may itself be coordinated through auxin transport.

In addition to its effect on growth rate, *JAG* also affects the extent of the distal organiser. Activity of auxin response marker *DR5::GFP* has a reduced and narrower distribution in the *jag* mutant while it has a broader distribution in the line that expresses *JAG* ectopically. Moreover in the *jag* mutant the distribution of the distal organiser (as marked by DR5 and PIN3) is more discontinuous, and this may account for the jagged or serrated shape of the distal petal margin. These results suggest that *JAG* may provide a distal identity to the petal, and as part of this identity, it increases the activity and distribution of the distal organiser.

One mechanism by which *JAG* may influence the activity of the distal organiser is through regulation of *PTL*, which we show is directly repressed by *JAG. PTL* functions upstream of auxin-mediated signalling in the control of petal development [30]. At early developmental stages, *PTL* is expressed at the inter-sepal zone of whorl 1 and is required to establish DR5 maxima at the presumptive petal-initiation sites in the adjacent whorl 2 [29],[30]. Thus, early *PTL* may be acting as a +organiser that helps to induce a −organiser at the tip of petal primordia. At later stages of petal development, *PTL* is expressed in the margin of the developing petal but excluded from the distal margin [44], where we propose the broad distal organiser acts. This exclusion may arise through *JAG* repressing *PTL* in this region, thus promoting −organiser activity.

The distinct morphogenetic pattern of leaves and petals in Arabidopsis most likely reflects an interaction between developmental and selective constraints [9]. Leaves need to be photosynthetically active from an early stage, particularly for an annual plant like Arabidopsis, which needs to grow and establish itself rapidly. Thus, it may be a selective advantage for leaf cells to begin differentiating and thus slow down growth as soon as they emerge and are exposed to light, which will happen first for cells at the tip [17]. Further increase in leaf size may then be generated by continued growth from the base. A further advantage of this growth mode is that it may allow plants to continue growing if the tip of the leaf is eaten by herbivores. By contrast, there is no early requirement for differentiation of the petal tip as these cells do not have a photosynthetic function, so growth along the proximodistal axis may be more uniformly distributed. The main function of petals is thought to be to provide a broad display that attracts pollinators, and this may be readily achieved through divergent orientations and enhanced distal growth. We show how both the leaf and petal growth modes can be accounted for by the same basic framework involving a polarity field with a specified pattern of growth rates parallel or perpendicular to it. The variation in form arises by simple modulations in the organisers of polarity and the distribution of growth rates. Thus the shape of petals and leaves can be seen to arise from modulations of a basic developmental process, which provides a space of possible forms, interacting with the selective constraints operating on each organ.

## Materials and Methods

### Plant Material and Growth Conditions

For petal clonal analysis and calculating growth curves, we used lines carrying *35S::lox-uidA-lox-GFP* and *hsp18.2::Cre* [36] in the Landsberg *erecta* (L-*er*) background. The *jag-1* mutant was originally isolated in Columbia (Col) genetic background [26] and was backcrossed into L-*er*. The *jag-2* and *jag-3 JAG::JAG:VENUS* lines were in L-*er* [32]. The *AP1::LhG4 OP::JAG OP::GFP* line (where the driver transactivated both JAG and a ER-targeted GFP) was in the L-*er* genetic background. To generate this line, *AP1:LhG4 OP::GFP* plants [58] were transformed with *OP::JAG*, in which the *JAG* cDNA was placed between the pOp operator [59] and the NOS terminator in the pPZP222 vector [60]. To generate the *JAG* ectopic line *AP1::LhG4 OP::JAG* (marked as *AP1>>JAG* in the text), lines without *OP::GFP* were selected. The transgenic line *35S::JAG:GR* in L-*er* background was generated and tested for complementation as described in [35].

The *DR5::GFP* [41],[42], *PIN1::PIN1:GFP* [41], and *PIN3::PIN3:GFP* [43] were in Col background. To introduce the different markers into the *jag-1* and *AP1>>JAG* backgrounds, we carried out the corresponding crosses.

For lines and crosses selection on media, seeds were surface-sterilized before plating on Petri dishes with solid Murashige and Skoog medium as described [61] and grown in controlled environment room at 20°C in long day conditions (8 h dark and 16 h light under fluorescent white light at a photon fluence rate of 100 µmol·m^−2^·s^−1^). When appropriate medium was supplemented with Kanamycin 50 µg/ml (for constructs *35S::lox-uidA-lox-GF*, *hsp18.2::Cre*, *AP1::LhG4*, *PIN1::PIN1:GFP*, and *PIN3::PIN3:GFP*), Gentamicin 100 µg/ml (for construct *OP::JAG*), or BASTA 5 µg/ml (for *jag-1* mutant and construct *OP::GFP*).

For petal measurements and petal clonal analysis, expression analysis, and ChIP experiments, seeds were directly sown on soil and plants grown until flowering in glasshouse controlled environment chambers at 20°C in long day conditions (8 h dark and 16 h light under fluorescent light at a photon fluence rate of 100 µmol·m^−2^·s^−1^) and 80% humidity.

### Petal Imaging

To measure petal width and length over time and to image different markers at various stages of petal development, petals were collected from flowers from various positions along the inflorescence axis. Given the hydrophobic nature of petals, they were flattened in 40% glycerol with 0–12% Tween-20 depending on petal developmental stage. Petals were imaged either on a Zeiss LSM 5 EXCITER Laser Scanning Confocal Microscope, or a Leica DM 6000 compound microscope and petal measurements were taken from 2D images using Fiji (http://fiji.sc).

For early stages of petal primordia (∼0 DAP), petals were imaged under the confocal microscope after staining with propidium iodide [62], and measurements were taken in 3D using VolViewer (http://www.uea.ac.uk/cmp/research/cmpbio/VolViewer).

### Petal Staging and Growth Curve

To provide a temporal framework for clone induction and visualisation, we determined the growth curves for wild-type petal width (at the petal's widest point). Flowers were collected from various positions along the inflorescence axis, starting with flower 1 (oldest flower) at the bottom of the inflorescence. To align flower stages to a common standard, we measured the rate at which flowers with visible petals (stage 13, as defined by [24]) emerge each day. This indicated that consecutive flowers are separated by about 11 h intervals (plastochron). Thus, a flower located N nodes above flower 1 should be approximately at the same stage as flower 1 would have been 11N h earlier. This allowed all flower stages to be aligned with flower 1 development. Thus, petals dissected from flowers along the inflorescence axis could be aligned with flower 1 petal development.

Growth rate in width (K_W_) was calculated as follows: (ln *W_n_*−ln *W_n-1_*)/T, in which *W_n_* is petal width at t_n_, *W_n-1_* petal width at t_n-1_, and T the time between t_n_ and t_n-1_.

Petal growth in width could be captured with a function with gradually decreasing growth rates. For this a logistic curve was fitted to the petal width data:
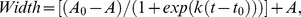
where *A* is the upper asymptote, *A_0_* is the lower asymptote, *t* is time (hours after sowing), *k* is the early exponential growth rate, and *t_0_* is the point of inflection. Parameters were estimated to be: *A* = 1.5 mm, *A_0_* = −0.08 mm, *k* = 0.9% h^−1^, and *t_0_* = 894 h.

We used 30 µm width as a convenient starting point for our developmental analysis and refer to this stage as 0 d after primordium formation (0 DAP). Petal primordia for flower 1 are about 30 µm wide at 14 d after sowing. At this stage, they have a simple primordium shape and are about 5–7 cells wide and 3–7 cells long. Petals took about 12 d to grow from 0 DAP to maturity. The logistic function was used to divide petal development into a series of 2-d intervals and estimate the average width of the petals at these times.

### Clonal Analysis

About 24 d after sowing (when flower 1, the oldest flower, was about to open; stage 13 as described by [24]), each plant inflorescence was heat shocked for ∼1.5 min, by submerging it into a water bath at 38°C. We used whole plants growing in soil in P40 trays. Previous to the HS, each plant pot was individually sealed with clean film to cover the entire rosette and leave out the inflorescence. After the HS, the clean film was removed and plants were taken back to the glasshouse. About 2–12 d afterward, petals were dissected out from flowers, flattened, and those with widths that correspond to 2–12 DAP imaged on a Zeiss LSM 5 EXCITER Laser Scanning Confocal Microscope, or a Leica DM 6000 compound microscope. When imaging, typically flower 1 (now identified as the flower at stage 13) and flower 2 (flower 1 node above flower 1) were around 10 DAP, flowers 5–6 ∼8 DAP, flowers 9–10 ∼6 DAP, flower 14 ∼4 DAP, and flowers 18–19 ∼2 DAP.

The Sector Analysis Toolbox was used to analyse clonal patterns in *Arabidopis* petals (http://www.uea.ac.uk/cmp/research/cmpbio/SectorAnalysisToolbox) as described in [15].

### Model Description

#### 1. Basic factors and functions

Growth patterns are determined by the pattern of factors distributed over the tissue, termed the canvas [39]. Factors have one value for each segment or vertex of the canvas and are denoted by capital letters in the text. In the equations, factors that propagate through the canvas are denoted by the bold letter **s** subscripted with the factor name, while those that are fixed to the canvas are denoted by **i** subscripted with the factor name. For instance, the mobile factor DGRAD is described by **s** *dgrad* in the equations.

Factors may promote growth rates through the linear function pro, defined as:
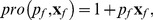
(1.1)where **x** *_f_* is a factor, F, and **x** denotes either **i** or **s**. *p_f_* is a promotion coefficient for that factor.

Values of all coefficients are given in Table 1. All models run from *t* = 24 h (0 DAP) to *t* = 312 h (12 DAP).

**Table 1:** Values of parameters used in the models.

Parameter	Model Description	Value
**PRN**		
Polariser		
*D_pol_*	POL diffusion constant	0.0005 mm^2^ h^−1^
*b_pol_*	Maximum POL levels	0.8
*μ_pol_*	POL decay rate	0.003 h^−1^
**KRN**		
Basic growth rates		
*b_Kper_*	Convergent/divergent	0.65% h^−1^
*b_Kpar_*	Convergent	1.7% h^−1^
*b_Kpar_*	Divergent	1.8% h^−1^
Promotion of K_per_ by DGRAD		
*p_dgrad_*	Convergent	1.7
*p_dgrad_*	Divergent	1.1

#### 2. Models

2D petal models were specified using the growing polarised tissue framework as implemented in the MATLAB application *GFtbox*. Full details of both are given in [39] and models can be downloaded with the *GFtbox* software from (https://www.uea.ac.uk/cmp/research/cmpbio/Gftbox). In this method an initial finite element mesh, also termed the canvas, is deformed during growth. The pattern of deformation depends on growth-modulating factors, whose initial distribution is established during setup. Factors have one value for each vertex and values between vertices are linearly interpolated across each finite element. In the models described here, the initial canvas is oriented with regard to the external *xy*-coordinate system such that the canvas base is parallel to the *x*-axis and the midline is parallel to the *y*-axis. The initial petal primordium canvas consists of 2,304 elements. Elements are not subdivided during the simulations. The initial canvas size is 30 µm in width and 30 µm in length (value of *y* = 0 µm at the base of the canvas).

Each model has two interconnected networks: the Polarity Regulatory Network (PRN) specifies tissue polarity and hence specified orientations of growth, and the Growth rate Regulatory Network (KRN) determines how factors influence specified growth rates.

In total, growth interactions are specified by three equations, one for the PRN and two for the KRN. These networks determine the specified polarity and growth fields across the canvas. Due to the connectedness of the canvas, this specified growth differs from the resultant growth by which the system is deformed. The time step of each model corresponds to 1 h of developmental time. Models take about 1 h to run on a dual core desktop computer.

These models involve growth orientations being established by POL propagation from a +organiser, which is expressed at the canvas base at the PROXORG region, towards a −organiser, which is expressed at the canvas tip at the DISTORG region. The gradient of POL defines the local polarity and hence local orientations of specified growth, which reorient with changes in the POL gradient.

#### PRN: Convergent model

An identity factor, PROXORG, is expressed at a level of 1 along the base of the canvas (green line in Figures 4A,D, 8A, 9A,B, and 10H,I) and 0 elsewhere.

The value of POL is fixed at a value of 0.8 (*b_pol_*), where PROXORG is expressed (i.e., where PROXORG = 1). POL diffuses according to the equation:
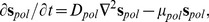
(2.1)where *D_pol_* is the diffusion rate and *μ_pol_* the decay rate of POL throughout the tissue. POL distribution is allowed to establish during the setup phase for 24 time steps before the commencement of growth.

POL continues to propagate and the polarity field readjusts according to POL levels throughout growth. The polarity field deforms during growth because changes in tissue geometry affect the way POL becomes distributed. In this model, the polarity pattern converges towards the tip.

#### PRN: Divergent model

PRN is the same as for the convergent model, but now an identity factor, DISTORG, is expressed at the level of 1 at the distal end of the canvas (cyan line in Figures 4D, 8A, 9A,B, and 10H,I), which consumes POL according to the equation:
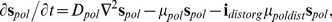
(2.2)where *μ_poldist_* is the decay rate of POL at the distal organiser region.

In this model, the polarity pattern diverges towards the distal margin of the canvas.

#### KRN: Convergent and divergent model

There is a basic specified growth rate parallel to the polarity gradient, K_par_ (*b_Kpar_*), and a basic specified growth rate perpendicular to the polarity gradient, K_per_ (*b_Kper_*) (values given in Table 1).

The value of K_per_ is promoted by DGRAD according to:
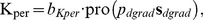
(2.3)where *p_dgrad_* is the promotion of K_per_ by DGRAD (values given in Table 1). DGRAD has a linear gradient across the canvas with the highest level of 1 at the tip plateau (as shown in bracketed region in Figures 4B,E, and 10H) and lowest level of 0 at the base. The gradient in DGRAD forms during setup (prior to the start of the growth simulation) and the value of DGRAD for each segment does not change as the line segments grow.

#### 3. Models parameter list

The models parameter list is presented in Table 1.

### Quantitative Reverse Transcription-Polymerase Chain Reaction (qRT-PCR)

To analyse *JAG* expression in *AP1>>JAG* plants with strong and intermediate phenotypes, seven inflorescence apices (flowers until stage 13) of the intermediate, wild type, and *jag-1* and nine inflorescence apices of the strong lines were collected in three biological replicates per line. To analyse *PTL* expression in the *35S::JAG:GR* line, for JAG:GR activation, inflorescences were dipped once into 0.015% Silwet L-77 (De Sangosse), 0.1% ethanol solution, supplemented with dexamethasone and cycloheximide as described in [35]. After 5 h in daylight conditions, inflorescence apices (flowers until stage 13) of 10 plants were collected per sample in three biological replicates per treatment. RNA extraction, reverse transcription, and qRT-PCR were performed as described in [35]. Quantitative PCR was performed in technical triplicates using primers JAG-RT_1-F and JAG-RT_1-R or PTL-RT_3-F and PTL-RT_3-R (Table S2) with the LightCycler 480 System and SYBR Green I (Roche). Data were normalized to *TUB4* expression (amplified with primers TUB4-RT_1-F, TUB4-RT_1-R; Table S2) as described in [63]. The Kruskal–Wallis test at the 5% alpha level was used for comparison of means between lines with different levels of *JAG* expression.

### Chromatin Immunoprecipitation (ChIP)

JAG:GR was activated as described above with 4 h incubation and ChIP was performed as described in [35]. Q-PCR was as described above with primers PTL-Ch_1-F/PTL-Ch_1-R and Mu-like_F/Mu-like_R (Mu-like transposon served as negative control) (Table S2).

### Accession Numbers

Sequence data can be found in the Arabidopsis Genome Initiative database under the accession numbers JAG (AT1G68480), PTL (AT5G03680), and TUB4 (AT1G04820).

## Supporting Information

Figure S1 AP1 promoter is active from early to late stages of petal development. Examples of early and late petals from an intermediate phenotype *AP1::LhG4 OP::JAG OP::GFP* plant. Width of petals shown, 55 and 470 µm.
(TIF)

Figure S2 *AP1>>JAG* lines phenotypes correlate with *JAG* expression levels. Expression levels (relative to the *TUB4* constitutive control) of *JAG* mRNA measured by qRT-PCR in inflorescences of wild type (WT), *AP1>>JAG* intermediate and strong phenotype plants, and *jag-1*. Error bars show the average and standard deviation of three biological replicates; letters a–c indicate that the expression level in the intermediate phenotype is significantly lower than in the strong phenotype (nonparametric Kruskal–Wallis test with a confidence level of 95%). Both phenotypes have significantly higher expression levels than the control. *JAG* transcripts were not detectable (n.d.) in the *jag-1* mutant.
(TIF)

Table S1 Epidermis cell division rates in the distal petal region are highest at early stages of petal development.
(DOC)

Table S2 List of oligos.
(DOC)

## Abbreviations

GPT-framework: Growing Polarised Tissue framework
K_par_: specified growth rate for a region, in the plane of the canvas, parallel to the local polarity
K_per_: specified growth rate for a region, in the plane of the canvas, perpendicular to the local polarity
KRN: Growth rate (K) Regulatory Network
+organiser: a region of the canvas that organises polarity with arrows pointing away from it
−organiser: a region of the canvas that organises polarity with arrows pointing towards it
POL: POLARISER (a signalling factor in the GPT-framework that is used to implement polarity propagation—the gradient of POL defines local polarity)
PRN: Polarity Regulatory Network

## References

[1] Turgeon R (1989) The sink-source transition in leaves. Annu Rev Plant Biol 40: 119–138.

[2] Pantin F, Simonneau T, Muller B (2012) Coming of leaf age: control of growth by hydraulics and metabolics during leaf ontogeny. New Phytol 196: 349–366. 22924516 10.1111/j.1469-8137.2012.04273.x

[3] Boege K, Marquis RJ (2005) Facing herbivory as you grow up: the ontogeny of resistance in plants. Trends Ecol Evol 20: 441–448. 16701415 10.1016/j.tree.2005.05.001

[4] McKey D (1974) Adaptive patterns in alkaloid physiology. Am Nat 108: 305–320.

[5] Rhoades DF (1979) Evolution of plant chemical defences against herbivory. In: Rosenthal GA, Janzen DH, editors. Herbivores: their interaction with secondary metabolites. Academic Press. pp. 3–54.

[6] Zangerl AR, Bazzaz FA (1992) Theory and pattern in plant defense allocation. In: Fritz RS, Simms EL, editors. Plant resistance to herbivores and pathogens. University of Chicago Press. pp. 363–391.

[7] Coen ES, Meyerowitz EM (1991) The war of the whorls: genetic interactions controlling flower development. Nature 353: 33–37. 10.1038/353031a0 1715520

[8] Goto K, Kyozuka J, Bowman JL (2001) Turning floral organs into leaves, leaves into floral organs. Curr Opin Genet Dev 11: 449–456. 11448632 10.1016/s0959-437x(00)00216-1

[9] Maynard-Smith J, Burian R, Kauffman S, Alberch P, Campbell J, et al (1985) Developmental constraints and evolution. Q Rev Biol 60: 265–287.

[10] Raff RA (1966) The shape of life: genes, development, and the evolution of animal form. Chicago: University of Chicago Press. 544 p.

[11] Prusinkiewicz P, Erasmus Y, Lane B, Harder LD, Coen E (2007) Evolution and development of inflorescence architectures. Science 316: 1452–1456. 17525303 10.1126/science.1140429

[12] Donnelly PM, Bonetta D, Tsukaya H, Dengler RE, Dengler NG (1999) Cell cycling and cell enlargement in developing leaves of Arabidopsis. Dev Biol 215: 407–419. 10545247 10.1006/dbio.1999.9443

[13] Tsukaya H (2005) Leaf shape: genetic controls and environmental factors. Int J Dev Biol 49: 547–555. 16096964 10.1387/ijdb.041921ht

[14] Barkoulas M, Galinha C, Grigg SP, Tsiantis M (2007) From genes to shape: regulatory interactions in leaf development. Curr Opin Plant Biol 10: 660–666. 17869569 10.1016/j.pbi.2007.07.012

[15] Kuchen EE, Fox S, Barbier de Reuille P, Kennaway R, Bensmihen S, et al (2012) Generation of leaf shape through early patterns of growth and tissue polarity. Science 335: 1092–1096. 22383846 10.1126/science.1214678

[16] Remmler L, Rolland-Lagan A-G (2012) Computational method for quantifying growth patterns at the adaxial leaf surface in three dimensions. Plant Physiol 159: 27–39. 22402928 10.1104/pp.112.194662 PMC3366717

[17] Andriankaja M, Dhondt S, De Bodt S, Vanhaeren H, Coppens F, et al (2012) Exit from proliferation during leaf development in Arabidopsis thaliana: a not-so-gradual process. Dev Cell 22: 64–78. 22227310 10.1016/j.devcel.2011.11.011

[18] Gonzalez N, Vanhaeren H, Inzé D (2012) Leaf size control: complex coordination of cell division and expansion. Trends in Plant Science 17: 332–340. 22401845 10.1016/j.tplants.2012.02.003

[19] Scarpella E, Marcos D, Friml J, Berleth T (2006) Control of leaf vascular patterning by polar auxin transport. Genes Dev 20: 1015–1027. 16618807 10.1101/gad.1402406 PMC1472298

[20] Wenzel CL, Schuetz M, Yu Q, Mattsson J (2007) Dynamics of MONOPTEROS and PIN-FORMED1 expression during leaf vein pattern formation in Arabidopsis thaliana. Plant J 49: 387–398. 17217464 10.1111/j.1365-313X.2006.02977.x

[21] Reinhardt D, Pesce E-R, Stieger P, Mandel T, Baltensperger K, et al (2003) Regulation of phyllotaxis by polar auxin transport. Nature 426: 255–260. 14628043 10.1038/nature02081

[22] Bilsborough GD, Runions A, Barkoulas M, Jenkins HW, Hasson A, et al (2011) Model for the regulation of Arabidopsis thaliana leaf margin development. Proc Natl Acad Sci U S A 108: 3424–3429. 21300866 10.1073/pnas.1015162108 PMC3044365

[23] Hill JP, Lord EM (1989) Floral development in Arabidopsis thaliana: a comparison of the wild type and the homeotic pistillata mutant. Can J Bot 67: 2922–2936.

[24] Smyth DR, Bowman JL, Meyerowitz EM (1990) Early flower development in Arabidopsis. Plant Cell 2: 755–767. 2152125 10.1105/tpc.2.8.755 PMC159928

[25] Pyke KA, Page AM (1998) Plastid ontogeny during petal development in Arabidopsis. Plant Physiol 116: 797–803. 9489024 10.1104/pp.116.2.797 PMC35139

[26] Dinneny JR, Yadegari R, Fischer RL, Yanofsky MF, Weigel D (2004) The role of JAGGED in shaping lateral organs. Development 131: 1101–1110. 14973282 10.1242/dev.00949

[27] Anastasiou E, Kenz S, Gerstung M, MacLean D, Timmer J, et al (2007) Control of plant organ size by KLUH/CYP78A5-dependent intercellular signaling. Developmental Cell 13: 843–856. 18061566 10.1016/j.devcel.2007.10.001

[28] Irish VF (2008) The Arabidopsis petal: a model for plant organogenesis. Trends Plant Sci 13: 430–436. 18603466 10.1016/j.tplants.2008.05.006

[29] Lampugnani ER, Kilinc A, Smyth DR (2012) PETAL LOSS is a boundary gene that inhibits growth between developing sepals in Arabidopsis thaliana. Plant J 71: 724–735. 22507233 10.1111/j.1365-313X.2012.05023.x

[30] Lampugnani ER, Kilinc A, Smyth DR (2013) Auxin controls petal initiation in Arabidopsis. Development 140: 185–194. 23175631 10.1242/dev.084582

[31] Disch S, Anastasiou E, Sharma VK, Laux T, Fletcher JC, et al (2006) The E3 ubiquitin Ligase BIG BROTHER controls Arabidopsis organ size in a dosage-dependent manner. Curr Biol 16: 272–279. 16461280 10.1016/j.cub.2005.12.026

[32] Ohno CK, Reddy GV, Heisler MGB, Meyerowitz EM (2004) The Arabidopsis JAGGED gene encodes a zinc finger protein that promotes leaf tissue development. Development 131: 1111–1122. 14973281 10.1242/dev.00991

[33] Hase Y, Tanaka A, Baba T, Watanabe H (2000) FRL1 is required for petal and sepal development in Arabidopsis. Plant J 24: 21–32. 11029701 10.1046/j.1365-313x.2000.00851.x

[34] Hase Y, Fujioka S, Yoshida S, Sun G, Umeda M, et al (2005) Ectopic endoreduplication caused by sterol alteration results in serrated petals in Arabidopsis. J Exp Bot 56: 1263–1268. 15737981 10.1093/jxb/eri122

[35] Schiessl K, Kausika S, Southam P, Bush M, Sablowski R (2012) JAGGED controls growth anisotropy and coordination between cell size and cell cycle during plant organogenesis. Curr Biol 22: 1739–1746. 22902754 10.1016/j.cub.2012.07.020 PMC3471073

[36] Gallois JL, Woodward C, Reddy GV, Sablowski R (2002) Combined SHOOT MERISTEMLESS and WUSCHEL trigger ectopic organogenesis in *Arabidopsis* . Development 129: 3207–3217. 12070095 10.1242/dev.129.13.3207

[37] Li S, Liu Y, Zheng L, Chen L, Li N, et al (2012) The plant-specific G protein γ subunit AGG3 influences organ size and shape in Arabidopsis thaliana. New Phytologist 194: 690–703. 22380792 10.1111/j.1469-8137.2012.04083.x

[38] Green AA, Kennaway JR, Hanna AI, Bangham JA, Coen E (2010) Genetic control of organ shape and tissue polarity. PLoS Biol 8: e1000537 doi:10.1371/journal.pbio.1000537. 21085690 10.1371/journal.pbio.1000537 PMC2976718

[39] Kennaway R, Coen E, Green A, Bangham A (2011) Generation of diverse biological forms through combinatorial interactions between tissue polarity and growth. PLoS Comput Biol 7: e1002071 doi:10.1371/journal.pcbi.1002071. 21698124 10.1371/journal.pcbi.1002071 PMC3116900

[40] Coen E, Rolland-Lagan AG, Matthews M, Bangham JA, Prusinkiewicz P (2004) The genetics of geometry. Proc Natl Acad Sci U S A 101: 4728. 14960734 10.1073/pnas.0306308101 PMC387316

[41] Benková E, Michniewicz M, Sauer M, Teichmann T, Seifertová D, et al (2003) Local, efflux-dependent auxin gradients as a common module for plant organ formation. Cell 115: 591–602. 14651850 10.1016/s0092-8674(03)00924-3

[42] Friml J, Vieten A, Sauer M, Weijers D, Schwarz H, et al (2003) Efflux-dependent auxin gradients establish the apical–basal axis of Arabidopsis. Nature 426: 147–153. 14614497 10.1038/nature02085

[43] Žádníková P, Petrášek J, Marhavý P, Raz V, Vandenbussche F, et al (2010) Role of PIN-mediated auxin efflux in apical hook development of Arabidopsis thaliana. Development 137: 607–617. 20110326 10.1242/dev.041277

[44] Brewer PB, Howles PA, Dorian K, Griffith ME, Ishida T, et al (2004) PETAL LOSS, a trihelix transcription factor gene, regulates perianth architecture in the Arabidopsis flower. Development 131: 4035–4045. 15269176 10.1242/dev.01279

[45] Petrášek J, Friml J (2009) Auxin transport routes in plant development. Development 136: 2675–2688. 19633168 10.1242/dev.030353

[46] Jönsson H, Heisler MG, Shapiro BE, Meyerowitz EM, Mjolsness E (2006) An auxin-driven polarized transport model for phyllotaxis. Proc Natl Acad Sci U S A 103: 1633–1638. 16415160 10.1073/pnas.0509839103 PMC1326488

[47] Smith RS, Guyomarc'h S, Mandel T, Reinhardt D, Kuhlemeier C, et al (2006) A plausible model of phyllotaxis. Proc Natl Acad Sci U S A 103: 1301–1306. 16432192 10.1073/pnas.0510457103 PMC1345713

[48] Sahlin P, Söderberg B, Jönsson H (2009) Regulated transport as a mechanism for pattern generation: capabilities for phyllotaxis and beyond. J Theor Biol 258: 60–70. 19490869 10.1016/j.jtbi.2009.01.019

[49] Rolland-Lagan A-G, Prusinkiewicz P (2005) Reviewing models of auxin canalization in the context of leaf vein pattern formation in Arabidopsis. Plant J 44: 854–865. 16297075 10.1111/j.1365-313X.2005.02581.x

[50] Stoma S, Lucas M, Chopard J, Schaedel M, Traas J, et al (2008) Flux-based transport enhancement as a plausible unifying mechanism for auxin transport in meristem development. PLoS Comput Biol 4: e1000207 doi:10.1371/journal.pcbi.1000207. 18974825 10.1371/journal.pcbi.1000207 PMC2565506

[51] Bayer EM, Smith RS, Mandel T, Nakayama N, Sauer M, et al (2009) Integration of transport-based models for phyllotaxis and midvein formation. Genes Dev 23: 373–384. 19204121 10.1101/gad.497009 PMC2648550

[52] Heisler MG, Hamant O, Krupinski P, Uyttewaal M, Ohno C, et al (2010) Alignment between PIN1 polarity and microtubule orientation in the shoot apical meristem reveals a tight coupling between morphogenesis and auxin transport. PLoS Biol 8: e1000516 doi:10.1371/journal.pbio.1000516. 20976043 10.1371/journal.pbio.1000516 PMC2957402

[53] Wabnik K, Kleine-Vehn J, Balla J, Sauer M, Naramoto S, et al. (2010) Emergence of tissue polarization from synergy of intracellular and extracellular auxin signaling. Mol Syst Biol 6.. 10.1038/msb.2010.103 PMC3018162 21179019

[54] Abley K, Barbier de Reuille P, Strutt D, Bangham A, Prusinkiewicz P, et al (2013) An intracellular partitioning-based framework for tissue cell polarity in plants and animals. Development 140 in press. doi:10.1242/dev.062984. 10.1242/dev.062984 23633507

[55] Roeder AHK, Chickarmane V, Cunha A, Obara B, Manjunath BS, et al (2010) Variability in the control of cell division underlies sepal epidermal patterning in Arabidopsis thaliana. PLoS Biol 8: e1000367 doi:10.1371/journal.pbio.1000367. 20485493 10.1371/journal.pbio.1000367 PMC2867943

[56] Baskin TI (2005) Anisotropic expansion of the plant cell wall. Annu Rev Cell Dev Biol 21: 203–222. 16212493 10.1146/annurev.cellbio.20.082503.103053

[57] Crowell EF, Gonneau M, Vernhettes S, Höfte H (2010) Regulation of anisotropic cell expansion in higher plants. C R Biologies 333: 320–324. 20371106 10.1016/j.crvi.2010.01.007

[58] Alvarez JP, Pekker I, Goldshmidt A, Blum E, Amsellem Z, et al (2006) Endogenous and synthetic microRNAs stimulate simultaneous, efficient, and localized regulation of multiple targets in diverse species. Plant Cell 18: 1134–1151. 16603651 10.1105/tpc.105.040725 PMC1456869

[59] Moore I, Gälweiler L, Grosskopf D, Schell J, Palme K (1998) A transcription activation system for regulated gene expression in transgenic plants. Proc Natl Acad Sci U S A 95: 376–381. 9419383 10.1073/pnas.95.1.376 PMC18229

[60] Hajdukiewicz P, Svab Z, Maliga P (1994) The small, versatile pPZP family of Agrobacterium binary vectors for plant transformation. Plant Mol Biol 25: 989–994. 7919218 10.1007/BF00014672

[61] Sauret-Güeto S, Calder G, Harberd NP (2012) Transient gibberellin application promotes Arabidopsis thaliana hypocotyl cell elongation without maintaining transverse orientation of microtubules on the outer tangential wall of epidermal cells. Plant J 69: 628–639. 21985616 10.1111/j.1365-313X.2011.04817.x

[62] Truernit E, Bauby H, Dubreucq B, Grandjean O, Runions J, et al (2008) High-resolution whole-mount imaging of three-dimensional tissue organization and gene expression enables the study of phloem development and structure in Arabidopsis. Plant Cell 20: 1494–1503. 18523061 10.1105/tpc.107.056069 PMC2483377

[63] Livak KJ, Schmittgen TD (2001) Analysis of relative gene expression data using real-time quantitative PCR and the 2(–Delta Delta C(T)) method. Methods 25: 402–408. 11846609 10.1006/meth.2001.1262

