# Supplementary figures and images for: JAGGED Controls Arabidopsis Petal Growth and Shape by Interacting with a Divergent Polarity Field
Source: PLoS Biol. 2013 Apr 30;11(4):e1001550. doi: 10.1371/journal.pbio.1001550 (PMC3641185; doi:10.1371/journal.pbio.1001550)

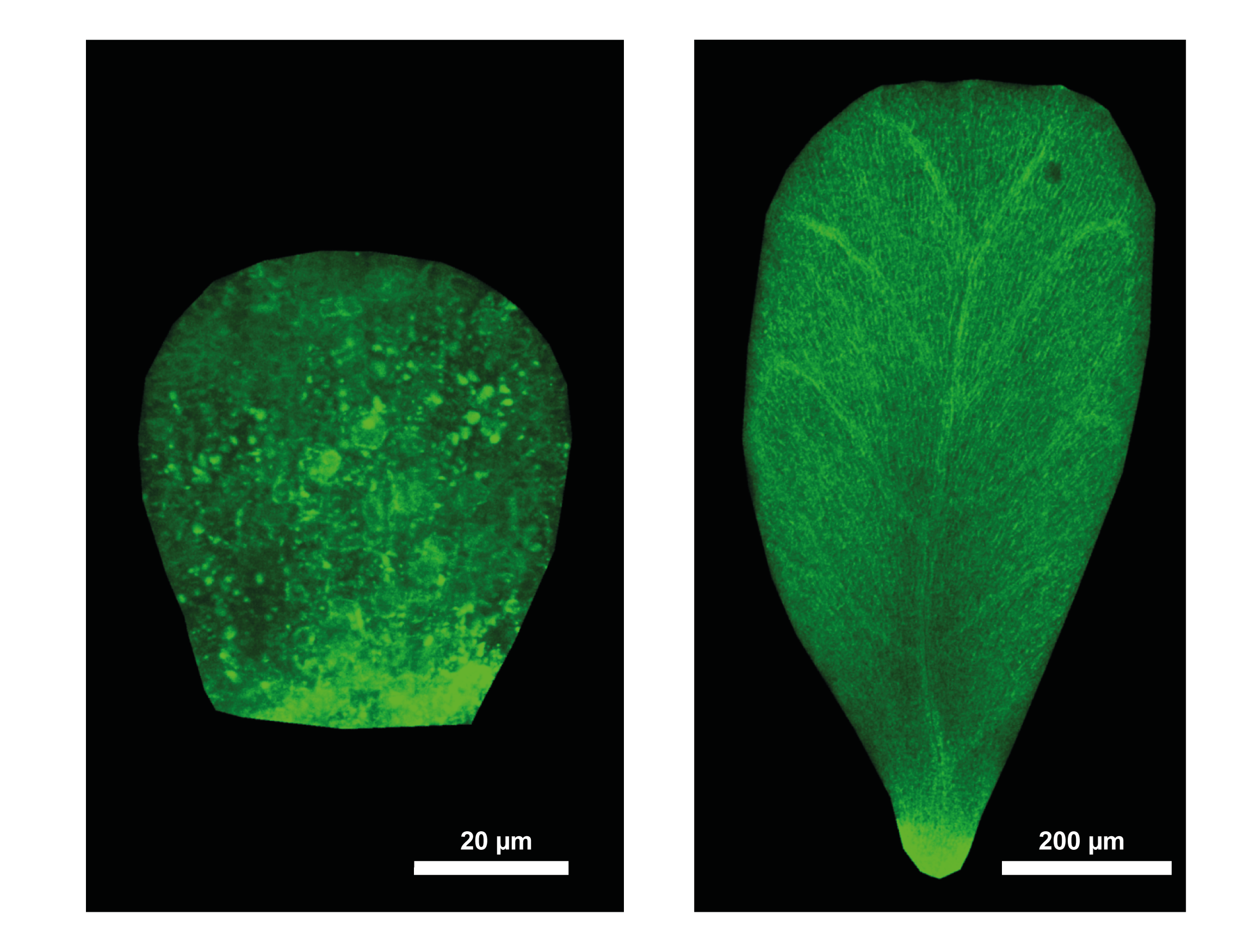

Supplement: Figure S1 — AP1 promoter is active from early to late stages of petal development. Examples of early and late petals from an intermediate phenotype AP1::LhG4 OP::JAG OP::GFP plant. Width of petals shown, 55 and 470 µm. (TIF) [file pbio.1001550.s001.tif]

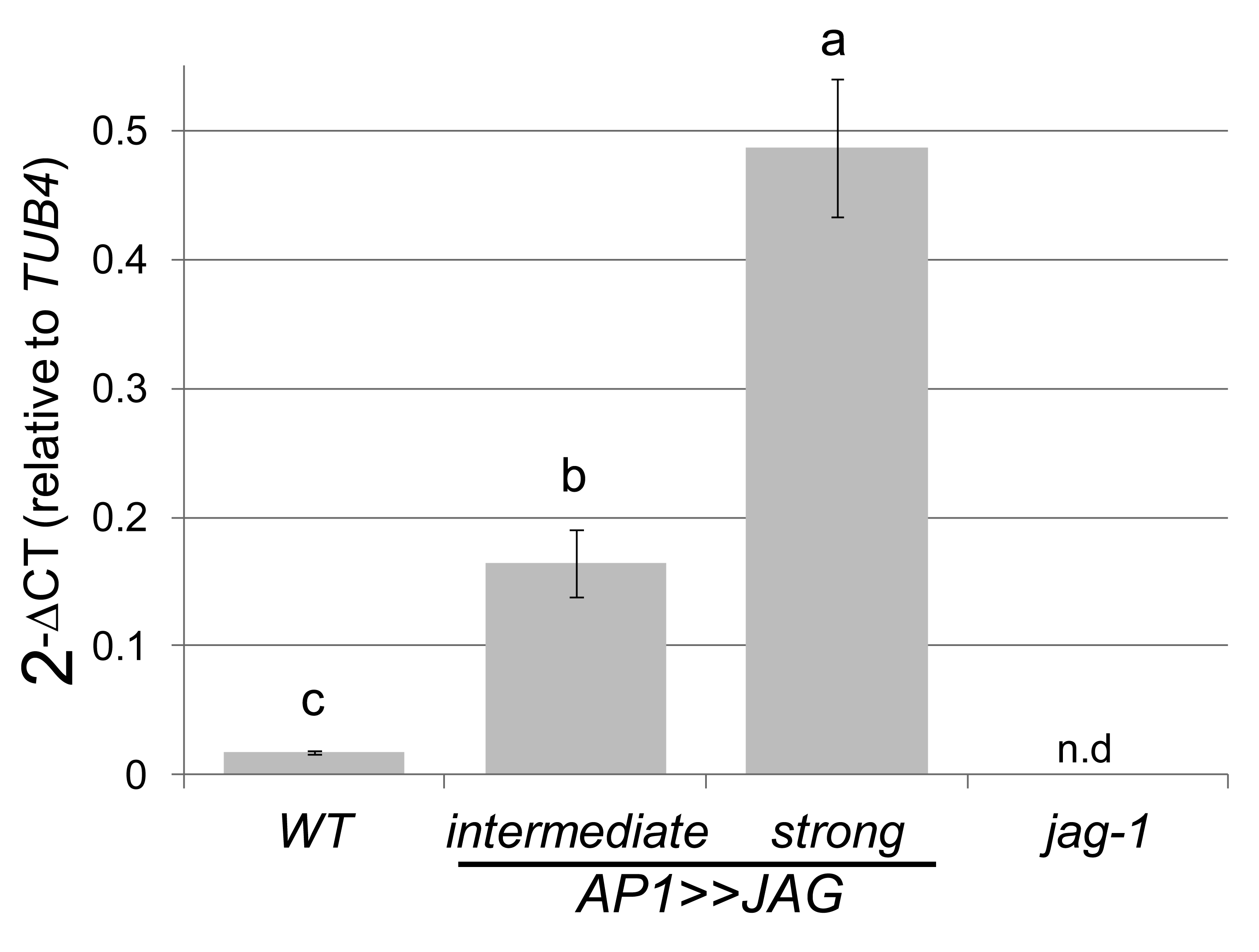

Supplement: Figure S2 — AP1>>JAG lines phenotypes correlate with JAG expression levels. Expression levels (relative to the TUB4 constitutive control) of JAG mRNA measured by qRT-PCR in inflorescences of wild type (WT), AP1>>JAG intermediate and strong phenotype plants, and jag-1. Error bars show the average and standard deviation of three biological replicates; letters a–c indicate that the expression level in the intermediate phenotype is significantly lower than in the strong phenotype (nonparametric Kruskal–Wallis test with a confidence level of 95%). Both phenotypes have significantly higher expression levels than the control. JAG transcripts were not detectable (n.d.) in the jag-1 mutant. (TIF) [file pbio.1001550.s002.tif]
