# Supplementary material for: JAGGED Controls Arabidopsis Petal Growth and Shape by Interacting with a Divergent Polarity Field
Source: PLoS Biol. 2013 Apr 30;11(4):e1001550. doi: 10.1371/journal.pbio.1001550 (PMC3641185; doi:10.1371/journal.pbio.1001550)
Supplement: Table S1 — Epidermis cell division rates in the distal petal region are highest at early stages of petal development. (DOC) [file pbio.1001550.s003.doc]

**Table S1**. **Epidermis cell divisions rates in the distal petal region are highest at early stages of petal development.**

|  | 0-2 DAP | 0-4 DAP | 0-8 DAP | 0-12 DAP |
| --- | --- | --- | --- | --- |
| % of final petal area | ~ 0.2% | ~ 2% | ~ 13% | ~ 100% |
| Increase in number of cells (from 1 cell at 0 DAP, if growth only due to cell divisions) | ~ 7 | ~ 50 | ~ 410 | ~ 3280 |
| Number of cells divisions for each DAP period (estimated from number of cells if growth only due to cell divisions) | ~ 2.8 | ~ 5.6 | ~ 8.7 | ~11.7 |
| Increase in number of cell based on cell numbers on distal clones | ~ 12 | ~ 30 | ~ 350 | ~ 540 |
| Number of cells divisions for each DAP period (estimated from number of cells in distal clones) | ~ 3.6 | ~ 5 | ~ 8.5 | ~ 9 |
