# Supplementary material for: JAGGED Controls Arabidopsis Petal Growth and Shape by Interacting with a Divergent Polarity Field
Source: PLoS Biol. 2013 Apr 30;11(4):e1001550. doi: 10.1371/journal.pbio.1001550 (PMC3641185; doi:10.1371/journal.pbio.1001550)
Supplement: Table S2 — List of oligos. (DOC) [file pbio.1001550.s004.doc]

**Table S2.** List of oligos

| JAG-RT_1_F | 5’ACATGAATCGCCACCGACAAGAG3’ |
| --- | --- |
| JAG-RT_1_R | 5’CGTTACGGTAGACCAATTGACGAG3’ |
| TUB4_F | 5’CTGTTTCCGTACCCTCAAGC3 |
| TUB4_R | 5’AGGGAAACGAAGACAGCAAG3’ |
| PTL_RT_3_F 5’GAAGGAAAAAGAGGAGTTGG3’ | 5’GAAGGAAAAAGAGGAGTTGG3’ |
| PTL_RT_3_R | 5’GTCAACTTCTCAAGCCAAAC3’ |
| PTL_Ch_1_F | 5’caaagacgagtgcctccatcta3’ |
| PTL_Ch_1_R | 5’tgttttgcaacatgtgtctagc3’ |
| Mu-like-F | 5’GATTTACAAGGAATCTGTTGGTGGT3’ |
| Mu-like-R | 5’CATAACATAGGTTTAGAGCATCTGC3’ |
